# Supplementary material for: Targeting lysosomal pH restores mitochondrial quality control in GBA1-mutant Parkinson’s disease
Source: Transl Neurodegener. 2026 Jun 17;15:27. doi: 10.1186/s40035-026-00559-z (PMC13277009; doi:10.1186/s40035-026-00559-z)
Supplement: Supplementary file 1 — Additional file 1. Table S1. List of cell lines used. Table S2. List of antibodies used in the study. Figure S1. Lysosomal abnormalities in GBA1-PD DA neurons and fibroblasts. Figure S2. Mitochondrial dysfunction in GBA1-PD DA neurons and fibroblasts. Figure S3. Mitophagy defects in GBA1-PD DA neurons and fibroblast. Figure S4. Effect of acidic NPs and rapamycin on GBA1-PD fibroblasts and DA neurons. [file 40035_2026_559_MOESM1_ESM.pdf]

Table S1 List of cell lines used

Cell Lines used	Cell Line ID	Age/Sex	Cell type	Mutation	Source	Ethics Committee Approval
CONTROL 1	CONTROL 1	77/Male	Fibroblasts	No Mutation	NHNN	PD Royal Free Ethics
N370S	ND34263	65/Male	Fibroblasts; source for NH50182 iPSCs	GBA1-N370S; Heterozygous	NIH-RUCDR	
CONTROL 2	CONTROL 2	55/Male	Fibroblasts; source for Control1 iPSCs	No Mutation	NHNN	PD Royal Free Ethics
E326K 1	Het1/ JS48753	58/Male	Fibroblasts; source for Het1/E326K 1 iPSCs	GBA1-E326K; Heterozygous	NHNN	PD Royal Free Ethics
E326K 2	ND41015	63/Male	Fibroblasts; source for ND50045 iPSCs	GBA1-E326K; Heterozygous	NIH-RUCDR	
Isogenic Control (IsoC)	NH50142	63/Male	iPSCs; Isogenic Control for ND50045	CRISPR corrected GBA1-E326K heterozygous mutation	NIH-RUCDR	
E326K	ND50045	63/Male	iPSCs	GBA1-E326K; Heterozygous	NIH-RUCDR	
Isogenic Control (IsoC)	NH50186	65/Male	iPSCs; Isogenic Control for NH50182	CRISPR corrected GBA1-N370S heterozygous mutation	NIH-RUCDR	
N370S	NH50182	65/Male	iPSCs	GBA1-N370S; Heterozygous	NIH-RUCDR	
Control	Control 1	55/Male	iPSCs	No Mutation	Derived from CONTROL 2 fibroblasts	PD Royal Free Ethics for CONTROL 2 fibroblasts
E326K 1	Het1/ JS48753	58/Male	iPSCs	GBA1-E326K; Heterozygous	Derived from Het1 fibroblasts	PD Royal Free Ethics for JS48753 fibroblasts
E326K 2	Het 2/ MB240649	63/Male	iPSCs	GBA1-E326K; Heterozygous	Derived from MB240649 fibroblasts	PD Royal Free Ethics (for MB240649 fibroblasts
Control	SFC156 (Control 2)	65/Male	iPSCs	No Mutation	EBiSC	
N370S 1	SFC834 (N370S1)	72/Male	iPSCs	GBA1-N370S; Heterozygous	EBiSC	
N370S 2	SFC848 (N370S2)	68/Male	iPSCs	GBA1-N370S; Heterozygous	EBiSC	

Table S2 List of antibodies used in the study

Name	Catalogue no.	Host	Company	Application	Concentration
ATP6V0D2	AB194557	Mouse	Abcam	Western blotting	1:1000
LAMP1 (H4A3)	sc-20011	Mouse	Santa Cruz	Western blotting, Immunofluorescence	1:500
GBA (c-term)	64171	Rabbit	Sigma	Western blotting	1:700
ATP6V1H	AB187706	Rabbit	Abcam	Western blotting	1:1000
ATP6V1A	199326	Rabbit	Abcam	Western blotting	1:1000
pMTOR	55365	Rabbit	Abcam	Western blotting	1:1000
MTOR	45175	Mouse	Cell Signalling	Western blotting	1:1000
ATG5 (D5F5U)	12994T	Rabbit	Cell Signalling	Western blotting	1:1000
P62	610833	Mouse	BD Biosystems	Western blotting	1:1000
LC3	L7543	Rabbit	Sigma	Western blotting	1:1000
B-ACTIN	ab8226	Mouse	Cell Signalling	Western blotting	1:5000
TOM20	ab186735	Rabbit	Abcam	Western blotting	1:1000
LC3	PM036	Rabbit	MBL Biosystems	Immunofluorescence	1:500
Citrate Synthetase	ab96600	Rabbit	Abcam	Immunofluorescence	1:200
OXPHOS Cocktail	45-8199	Mouse	Thermoscientific	Western blotting	1:1000
Alexa Fluor 488	A-11008	Goat	Thermoscientific	Immunofluorescence	1:1000
Alexa Fluor 594	A-11012	Goat	Thermoscientific	Immunofluorescence	1:1000
Alexa Fluor 647	A-21235	Goat	Thermoscientific	Immunofluorescence	1:1000
IRDye® 680RD Goat anti-Mouse	926-68070	Goat	Li-COR Biosciences	Western blotting	1:10000
IRDye® 800CW Goat anti-Rabbit IgG	926-32211	Goat	Li-COR Biosciences	Western blotting	1:10000

Supplementary Figure 1

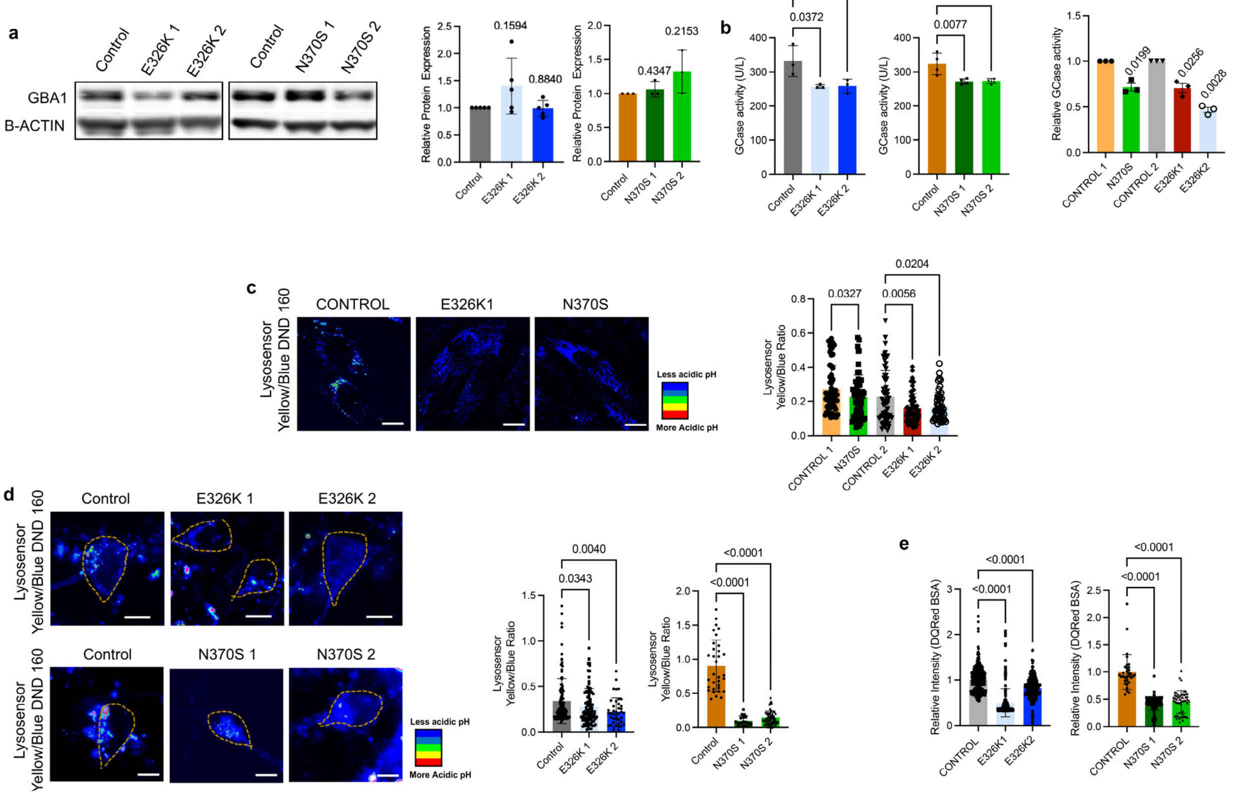

Figure S1: Lysosomal abnormalities in GBA1-PD DA neurons and fibroblasts. a GBA1 protein expression and subsequent quantification of GBA1 levels in control and GBA1-E326K and N370S DA neurons. **b** GCase activity in GBA1-E326K and GBA1-N370S DA neurons and fibroblasts. **c, d** Representative ratioed images of GBA1-E326K and GBA1-N370S fibroblasts (**c**) and DA neurons (**d**) stained with Lysosensor Yellow/Blue DND 160, and subsequent quantification of fluorescence ratio measuring lysosomal pH. Scale – 10 μm. **e** Histogram representing quantification of DQ-Red BSA intensity in GBA1-E326K and GBA1-N370S and control DA neurons, indicating lysosomal proteolytic activity. Data presented as mean±SD; one-sample Wilcoxon rank t-test or One-way ANOVA with Kruskal-Wallis and Dunn's multiple comparisons test or Holm-Šídák's multiple comparisons test. *P* values are noted on the graphs. The statistical tests, corresponding effect sizes, and confidence intervals for each graph are in Additional file 2**.**

Supplementary Figure 2

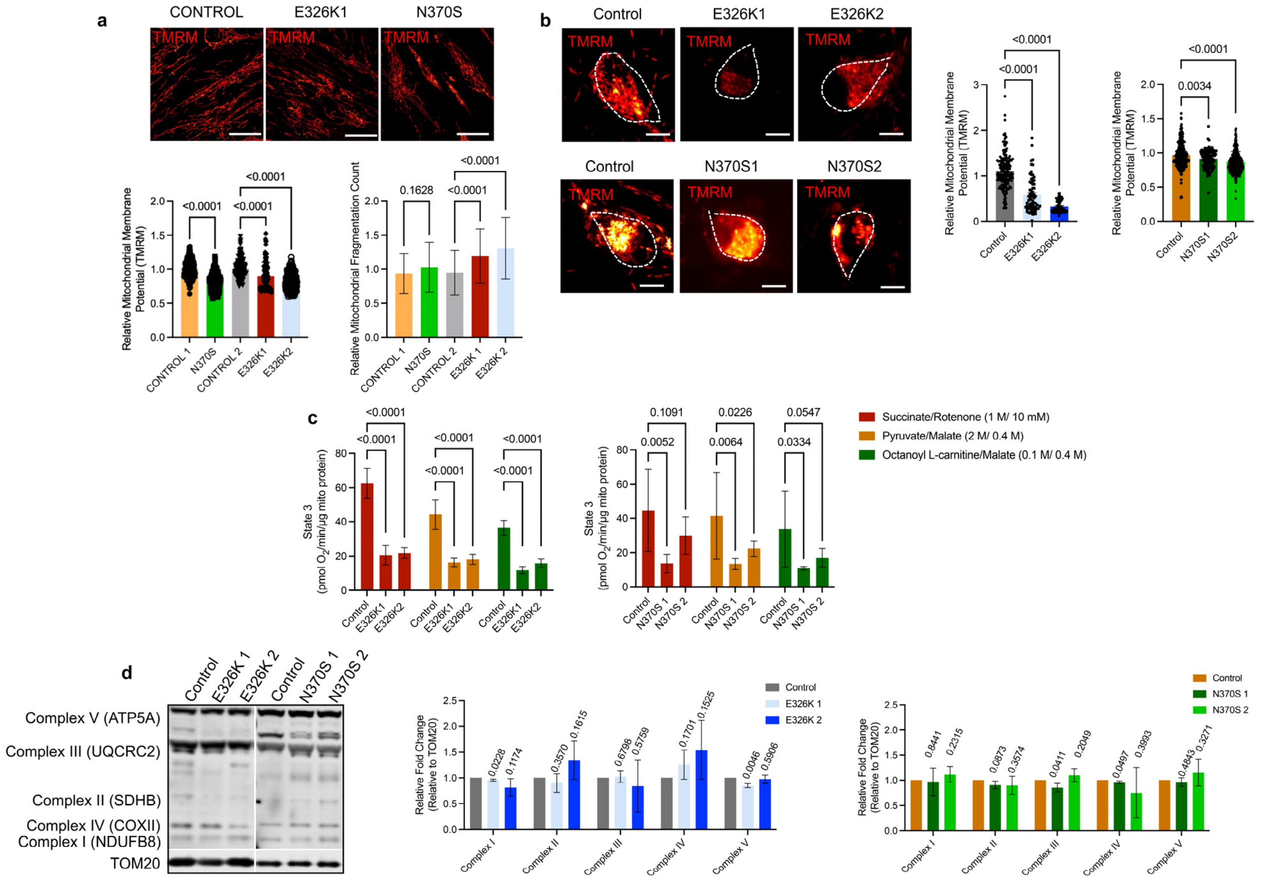

Figure S2: Mitochondrial dysfunction in GBA1-PD DA neurons and fibroblasts. a, b Representative images of GBA1-E326K and GBA1-N370S fibroblasts (Scale - 30 µm) (**a**) and DA neurons (**b**) along with controls, stained with TMRM to measure changes in ΔΨm (**a, b**) and the mitochondrial fragmentation count (**a**) (Scale – 10 µm). **c** Histograms representing State 3 (ATP Production) in mitochondria isolated from GBA1-E326K and GBA1-N370S-DA neurons and controls, as measured by Seahorse assay. **d** Representative images of western blots probed for OXPHOS complex proteins and subsequent quantification of protein expression levels in GBA1 E326K and N370S DA neurons. Data represented as mean±SD; Statistics: one-sample Wilcoxon rank t-test or One-way ANOVA with Kruskal-Wallis and Dunn's, Holm-Šidák or Šídák's multiple comparisons test or Ordinary two-way ANOVA with Tukey's multiple comparisons test with single pooled variance. *P* values are noted on the graphs. The statistical tests, corresponding effect sizes, and confidence intervals for each graph are in Additional file 2.

Supplementary Figure 3

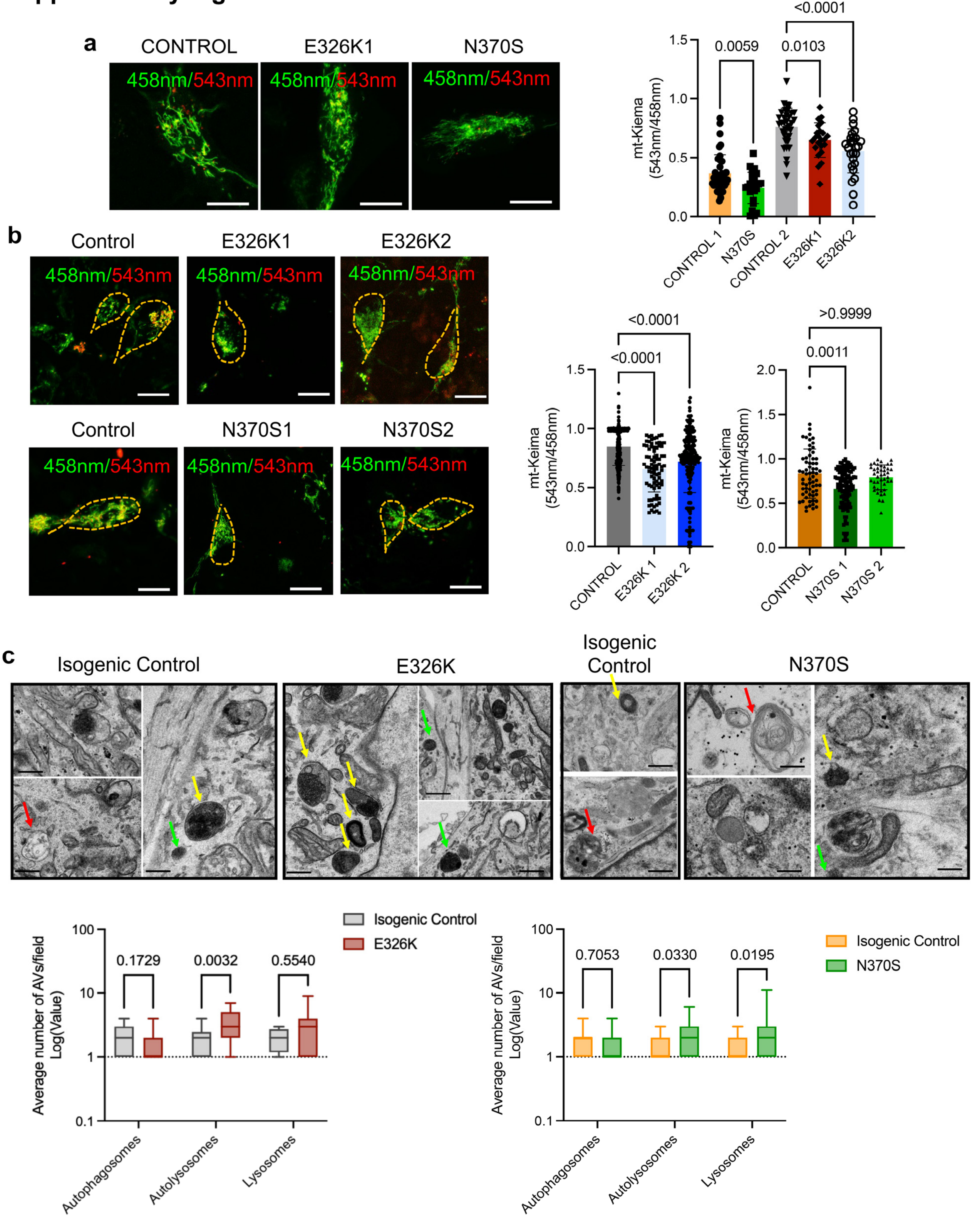

Figure S3: Mitophagy defects in GBA1-PD DA neurons and fibroblast. Representative ratioed images of control and GBA1-E326K and GBA1-N370S fibroblasts (Scale bar, 30 μm) (**a**) and DA neurons (**b**) transduced with mt-Keima plasmid and imaged imaged with excitation at 458 and 543nm and subsequent quantification of the ratio of signals at 543/458nm , respectively (Scale bar, 10 μm). **c** Representative TEM images of GBA1-E326K and GBA1-N370S DA neurons and respective isogenic controls depicting autophagic vesicles and-Box-and-whisker plots showing the minimum, maximum, median, and interquartile range of the log(1+Y) values. Yellow Arrows indicate autolysosomes, red arrows indicate autophagosomes, and green arrows indicate lysosomes in the cells. Scale bar, 0.5 μm. Data represented as mean±SD; Statistics: One-way ANOVA with Kruskal-Wallis and Dunn's multiple comparisons test or Holm-Šidák multiple comparisons test or Ordinary

two-way ANOVA with Šidák multiple comparisons test. *P* values are noted on the graphs. The statistical tests, corresponding effect sizes, and confidence intervals for each graph are in Additional file 2.

Supplementary Figure 4

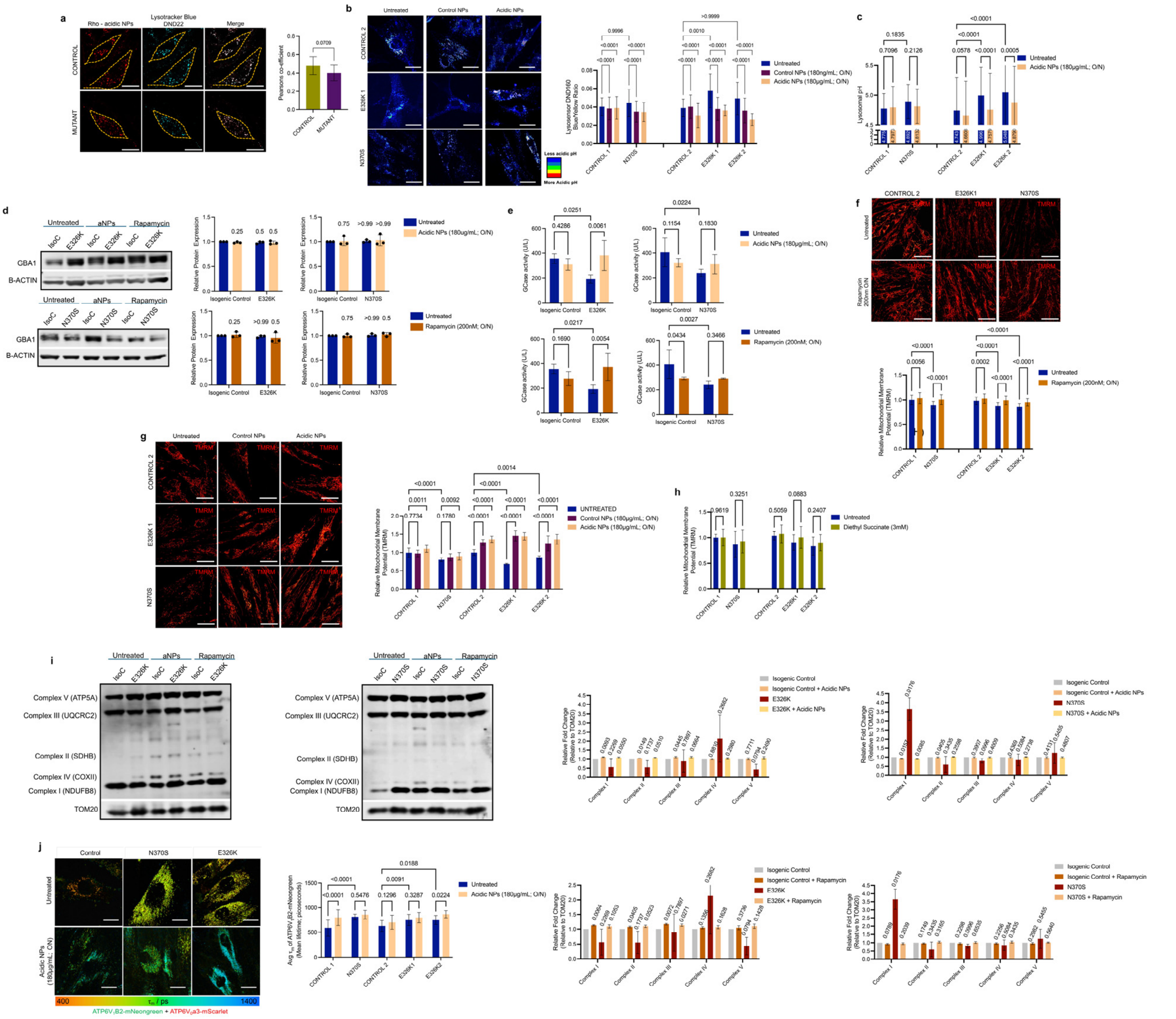

Figure S4: Effect of acidic NPs and rapamycin on GBA1-–PD fibroblasts and DA neurons: a Representative Confocal images of control and mutant fibroblasts treated with Rhodamine-tagged acidic NPs (Rhod-acidic NPs) and stained with Lysotracker Blue and subsequent quantification of colocalisation coefficient. Scale - 30µm. **b** Representative images of control and GBA1-E326K and GBA1-N370S fibroblasts treated with control and acidic NPs and stained with the ratiometric Lysosensor Yellow/Blue DND160 subsequent quantification of fluorescence ratio to measure lysosomal pH. Scale – 30 µm. **c** Calibration of lysosomal pH in *GBA1* mutant fibroblasts treated with and without acidic nanoparticles. **d** Representative images of western blots and quantification of GBA1 protein levels in GBA1-E326K and GBA1-N370S DA neurons and respective isogenic controls treated with acidic NPs or rapamycin and probed for GBA1 and B-ACTIN. **e** GCase activity in GBA1-E326K and GBA1-N370S DA neurons and respective isogenic controls treated with Acidic NPs/rapamycin. Images representing rapamycin-treated (**f**) and Acidic NPs treated (**g**) control and GBA1-E326K and GBA1-N370S fibroblasts, and stained with TMRM and subsequent quantification of ΔΨm. Scale - 30µm. Histogram representing quantification of mitochondrial membrane potential in *GBA1* mutant and control fibroblasts upon 3mM Diethyl succinate treatment for 30 minutes (**h**). **i** Representative images of western blots and subsequent quantification of OXPHOS complex proteins in GBA1 E326K and N370S DA neurons and respective isogenic controls treated with Acidic NPs and rapamycin. **j** Representative FLIM images of ATP6V$_1$B2-mNeongreen in Control and GBA1-PD fibroblasts co-transfected with ATP6V$_0$a3-mScarlet and treated with/without acidic NPs and subsequent quantification of mean lifetime (τ_m) of ATP6V$_1$B2. Scale – 30 µm. Data represent as mean±SD; Mann-Whitney's test, one-sample Wilcoxon rank t-test or Ordinary two-way ANOVA with Tukey's multiple comparisons test or Uncorrected Fisher's LSD with single pooled variance. *P*-values are noted on the graphs. The statistical tests, corresponding effect sizes, and confidence intervals for each graph are in Additional file 2
