# Supplementary material for: Targeting lysosomal pH restores mitochondrial quality control in GBA1-mutant Parkinson’s disease
Source: Transl Neurodegener. 2026 Jun 17;15:27. doi: 10.1186/s40035-026-00559-z (PMC13277009; doi:10.1186/s40035-026-00559-z)

Figure 1 Ai

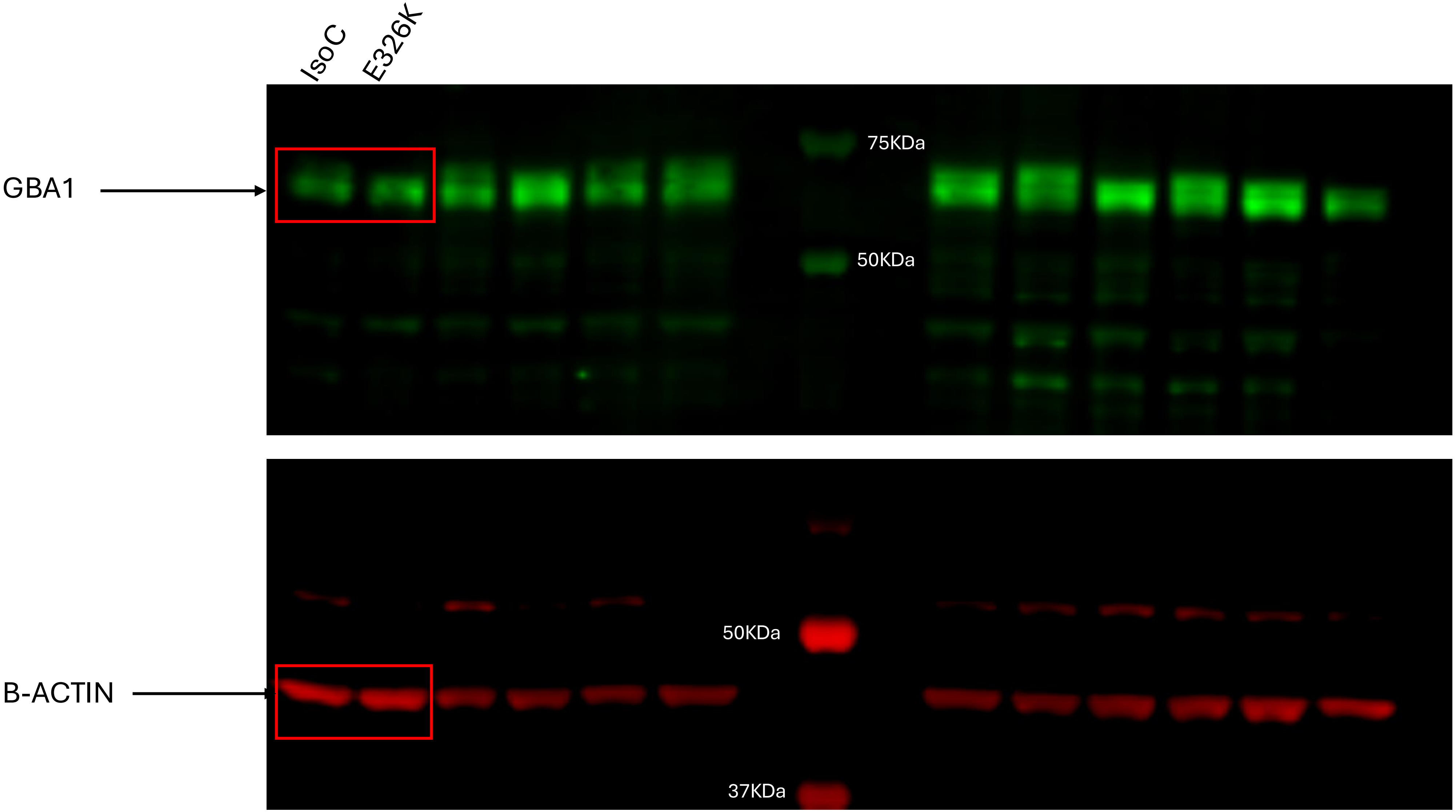

Figure 1 Ai

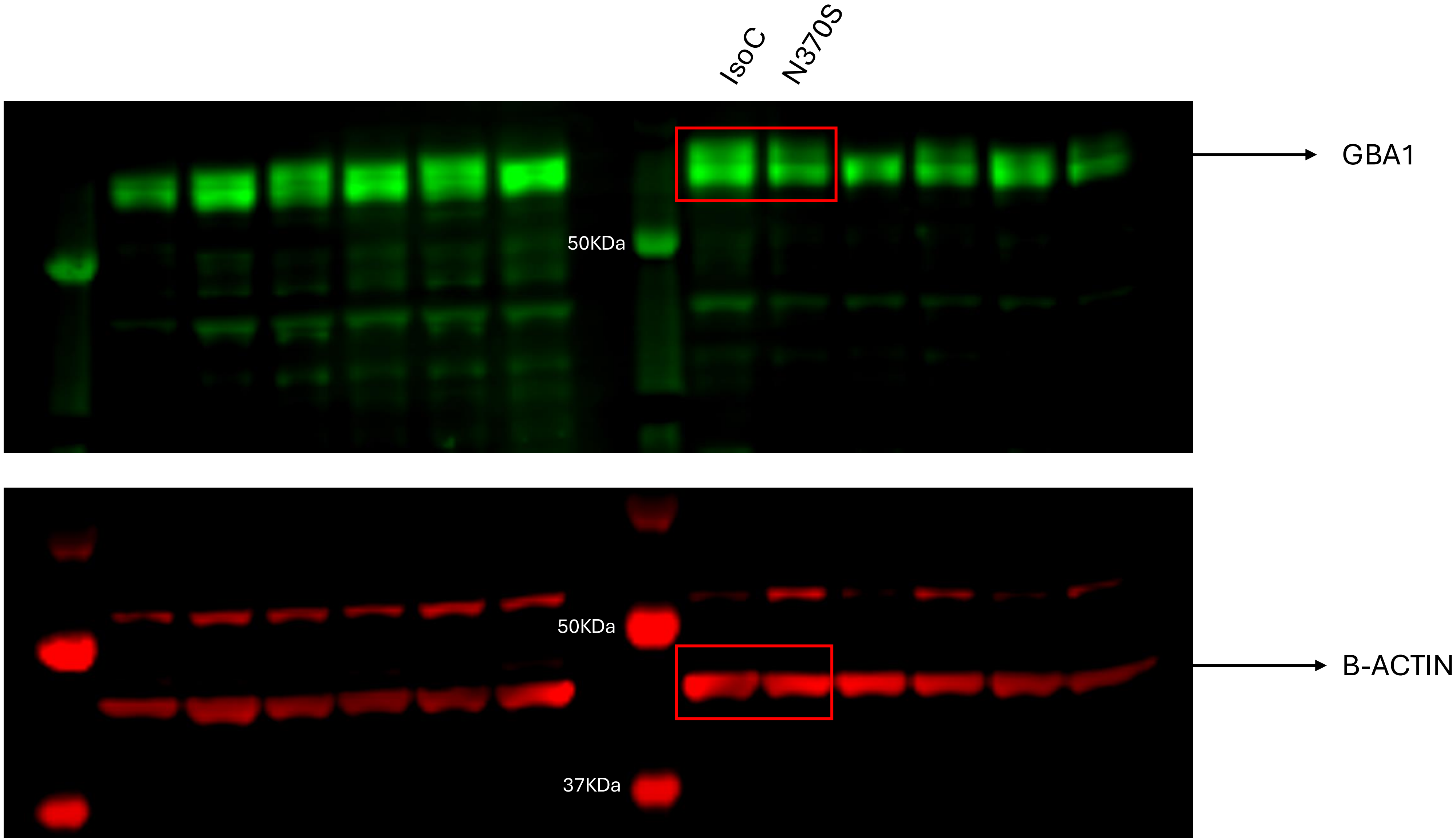

Figure 2 Ei

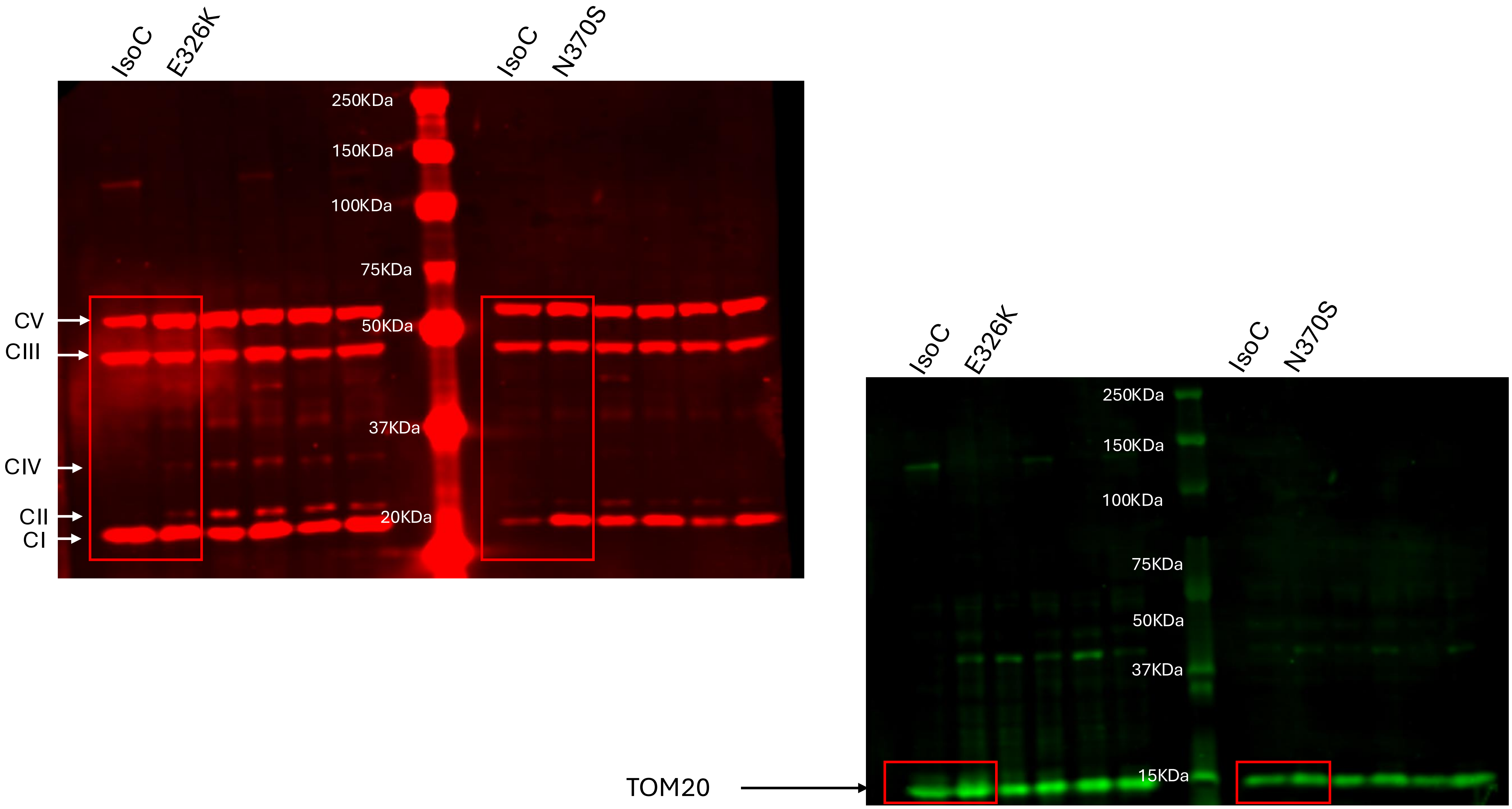
IsoC
E326K
IsoC
N370S
250KDa
150KDa
100KDa
75KDa
50KDa
37KDa
20KDa
CV
CIII
CIV
CII
CI
IsoC
E326K
IsoC
N370S
250KDa
150KDa
100KDa
75KDa
50KDa
37KDa
15KDa
TOM20

Figure 3 Bi)

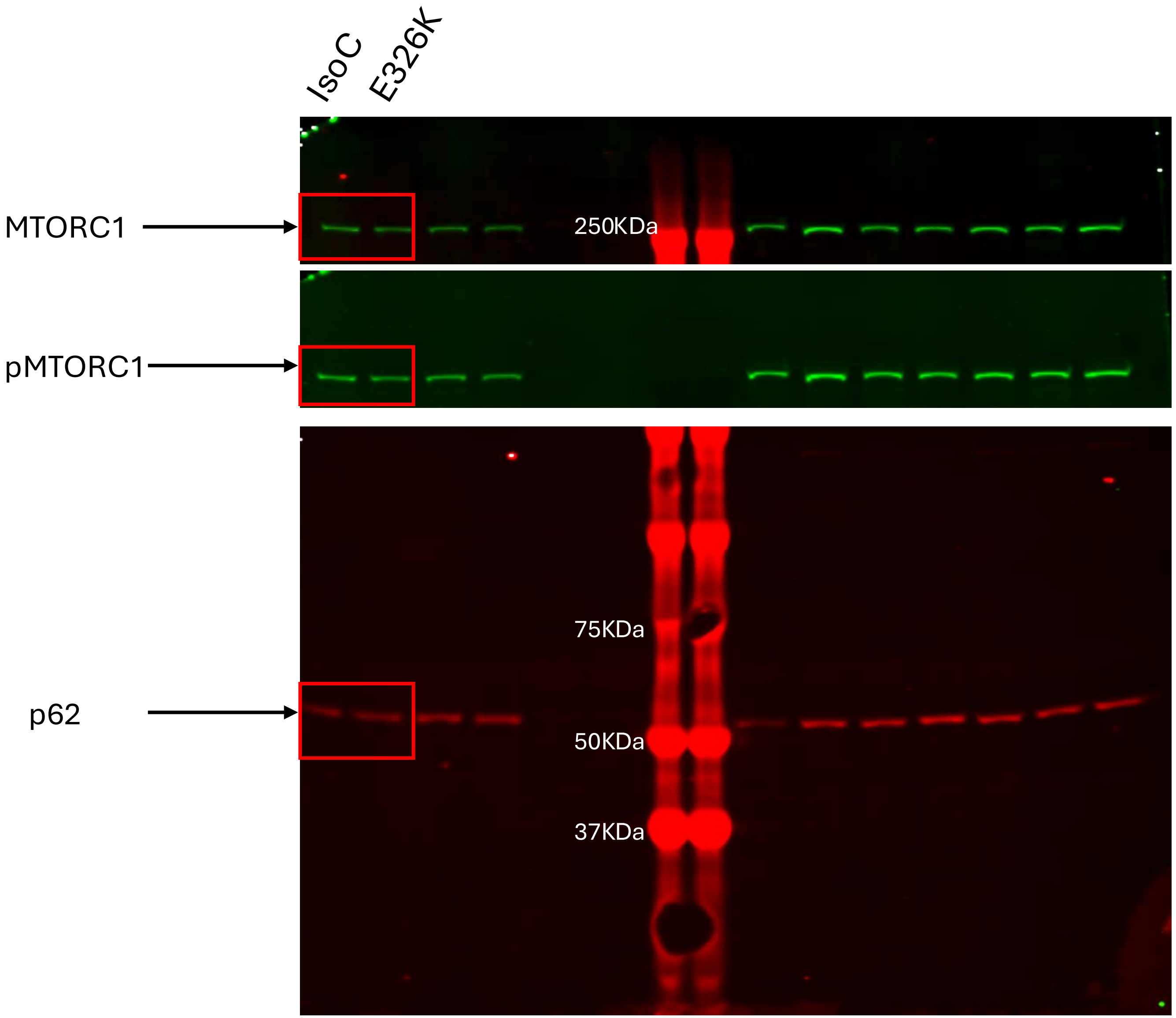

Figure 3 Bi)

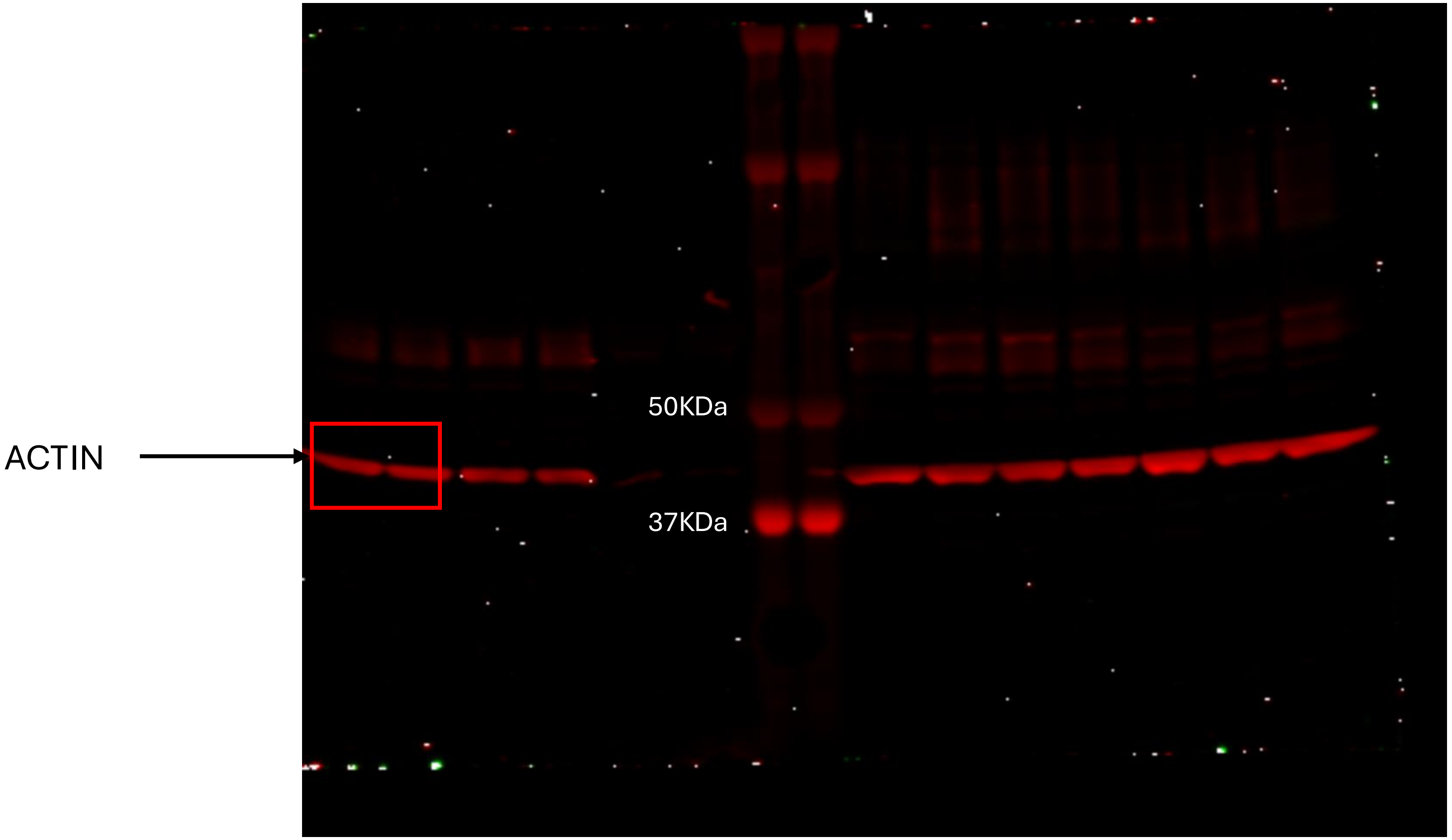

Figure 3 Bi)

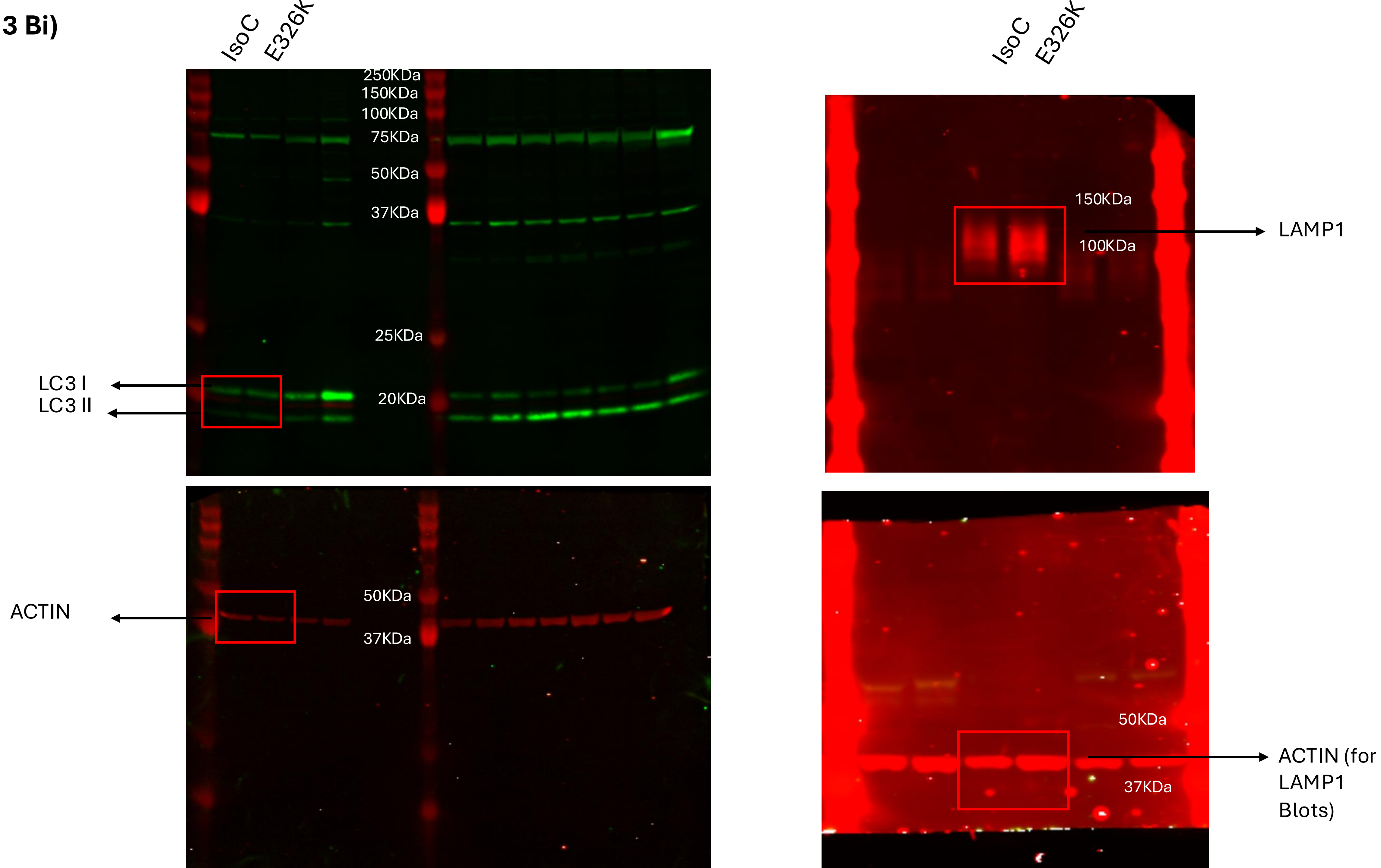

IsoC
E326K
250KDa
150KDa
100KDa
75KDa
50KDa
37KDa
25KDa
20KDa
LC3 I
LC3 II
ACTIN
50KDa
37KDa
IsoC
E326K
150KDa
100KDa
LAMP1
50KDa
37KDa
ACTIN (for LAMP1 Blots)

Figure 3 Bi)

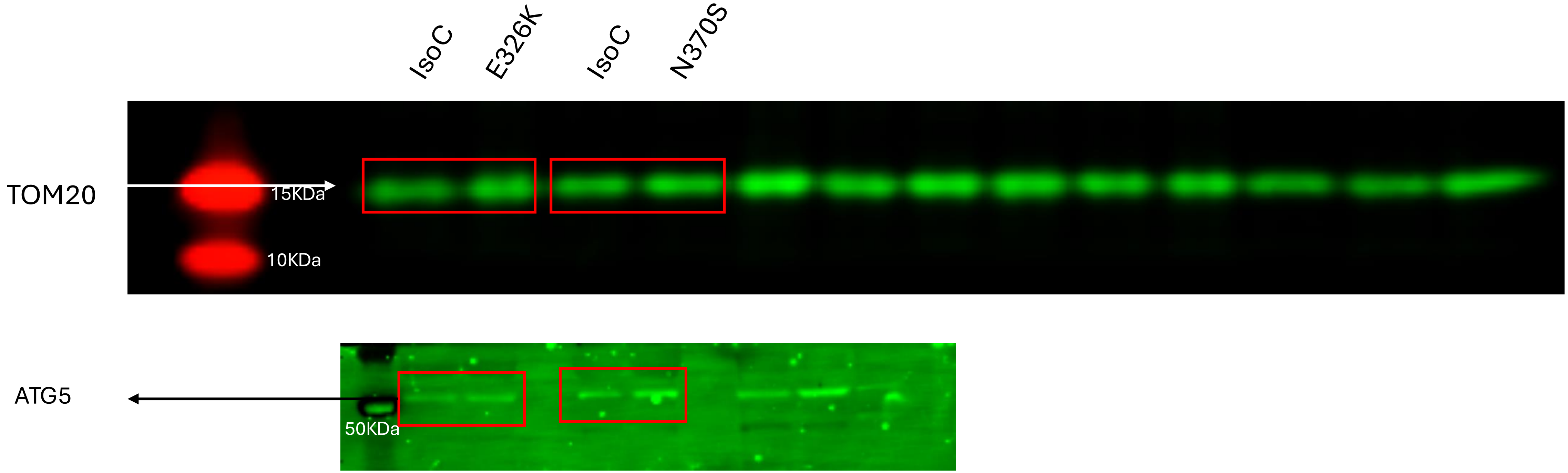

Figure 3 Bi)

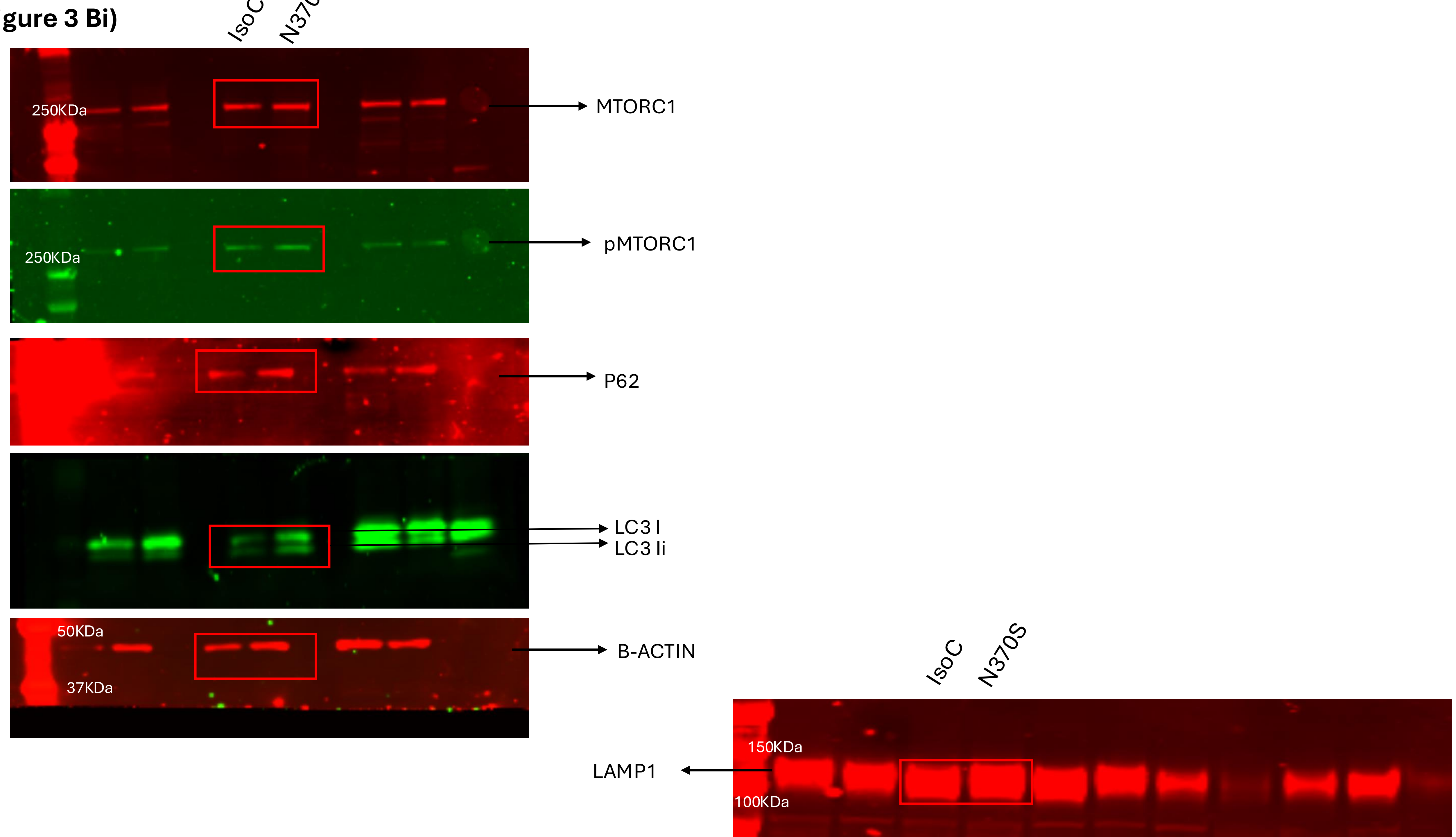

IsoC
N370
250KDa
MTORC1
250KDa
pMTORC1
P62
LC3 I
LC3 Ii
50KDa
37KDa
B-ACTIN
IsoC
N370S
150KDa
100KDa
LAMP1

Figure 4 Ai)

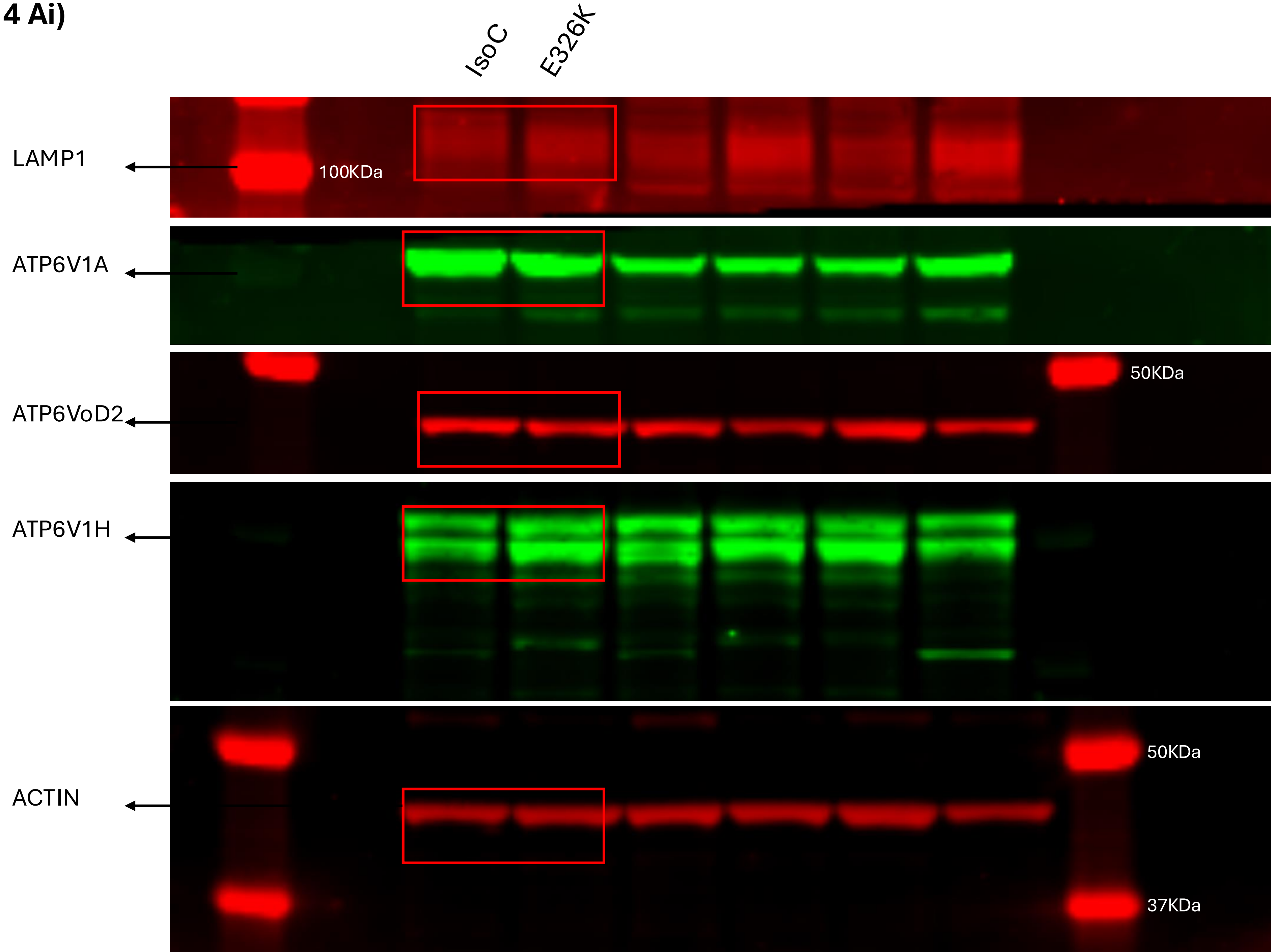
IsoC
E326K
LAMP1
100KDa
ATP6V1A
ATP6VoD2
50KDa
ATP6V1H
ACTIN
50KDa
37KDa

Figure 4 Ai)

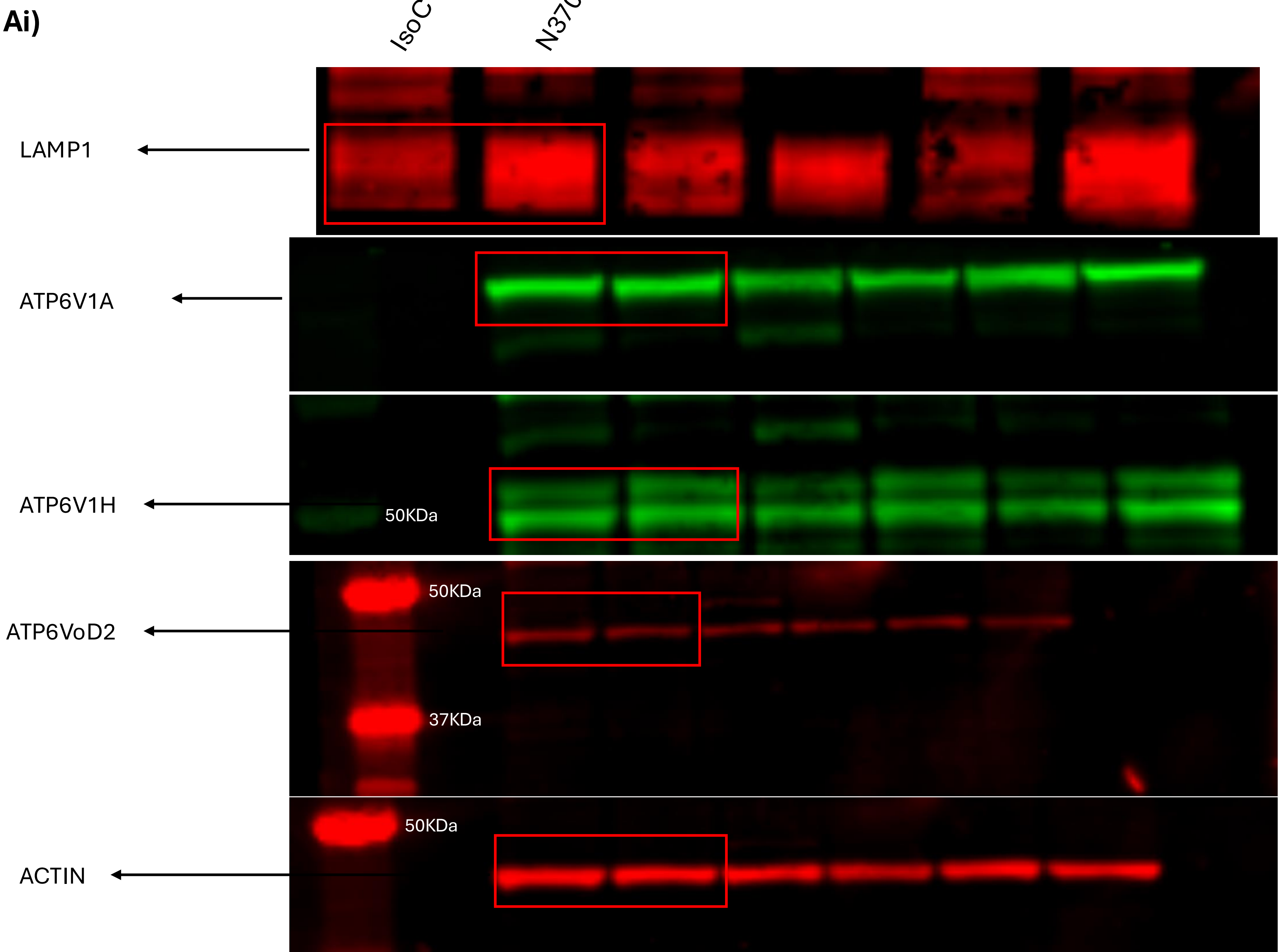
IsoC
N37
LAMP1
ATP6V1A
ATP6V1H
50KDa
50KDa
ATP6VoD2
37KDa
50KDa
ACTIN

Figure 4 Bi)

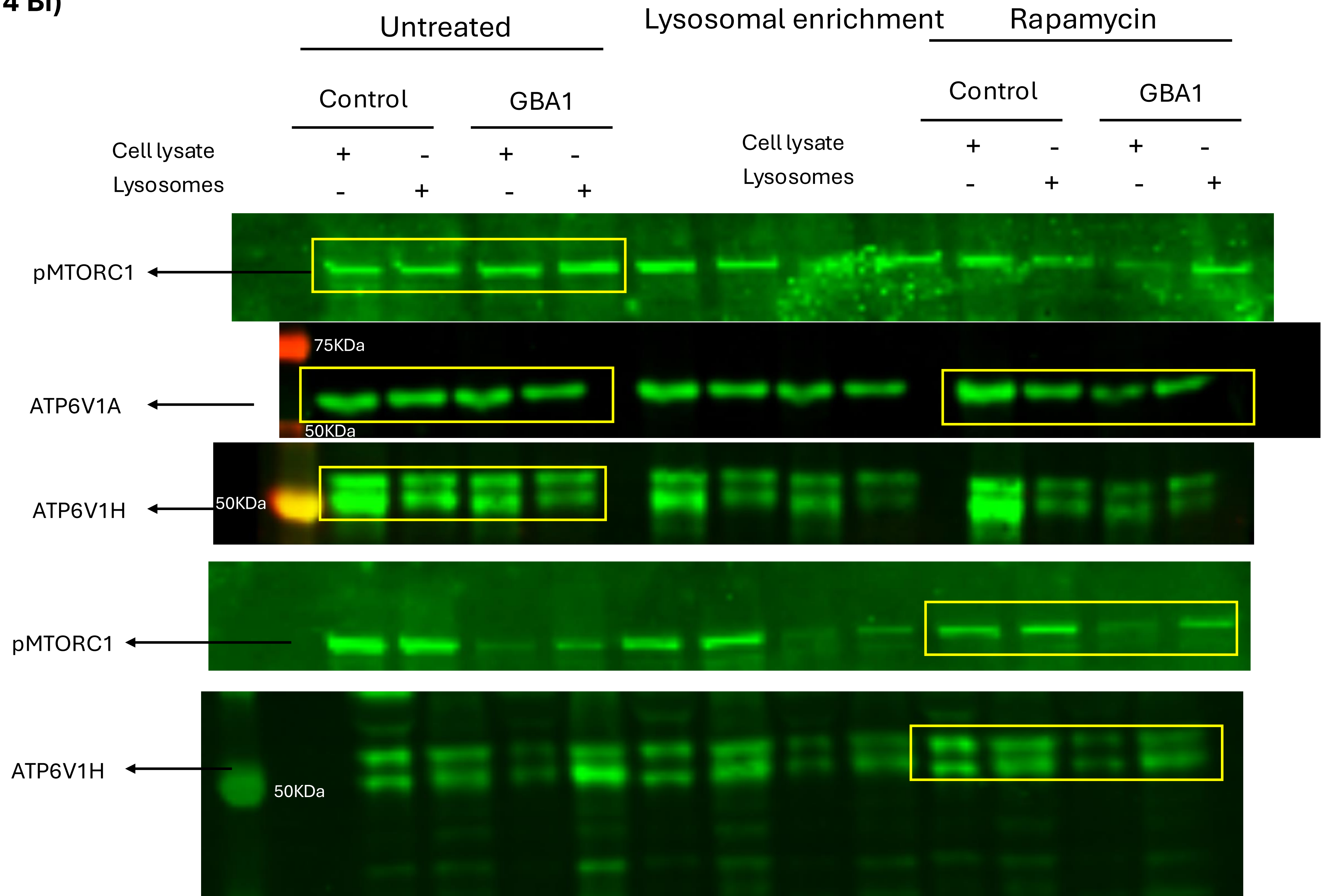

Untreated
Lysosomal enrichment
Rapamycin
Control
GBA1
Control
GBA1
Cell lysate
Lysosomes
Cell lysate
Lysosomes
+ - + -
- + - +
+ - + -
- + - +
pMTORC1
ATP6V1A
75KDa
50KDa
ATP6V1H
50KDa
pMTORC1
ATP6V1H
50KDa

Figure 4 Bi)

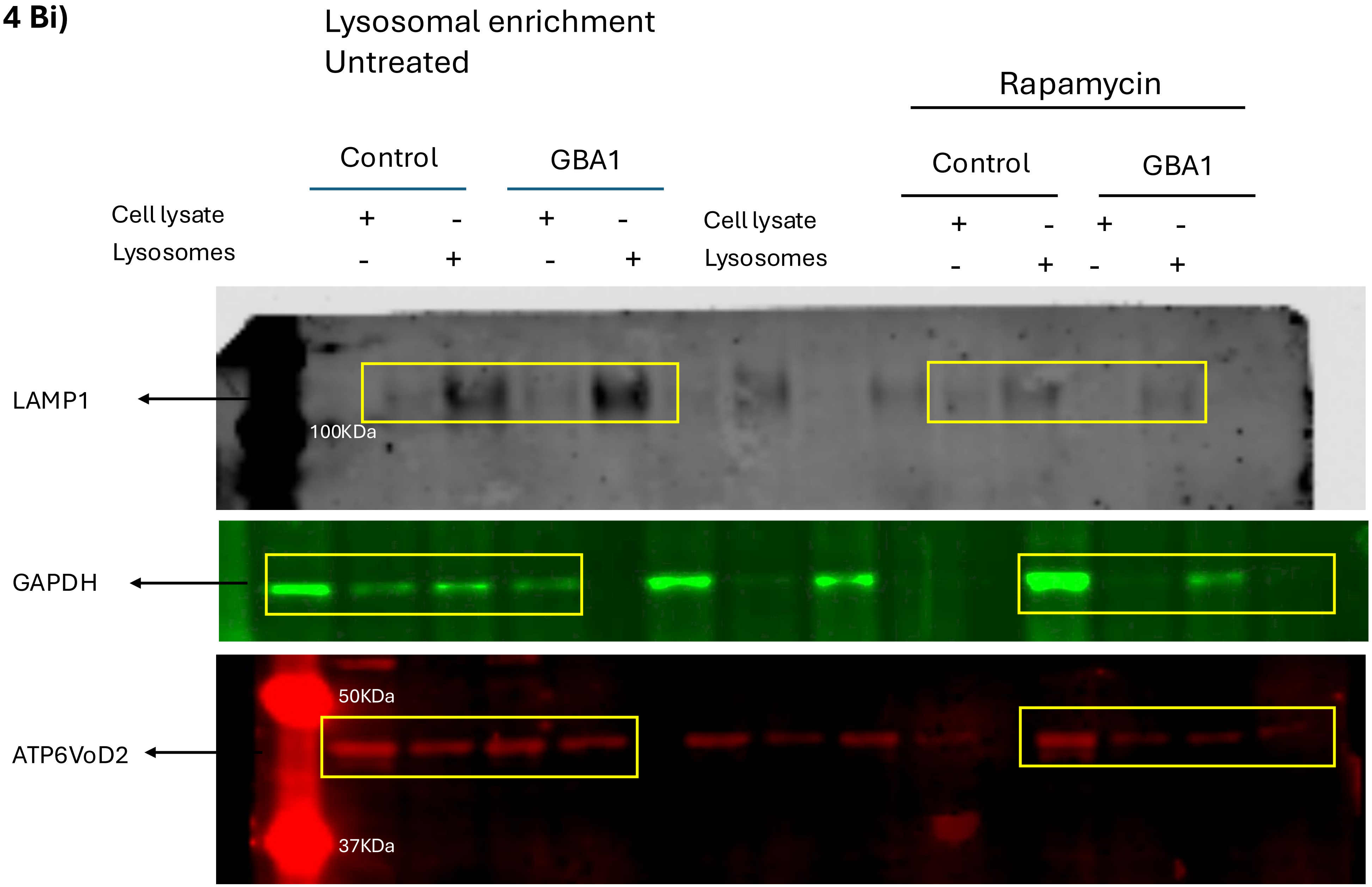
Lysosomal enrichment
Untreated
Rapamycin
Control
GBA1
Control
GBA1
Cell lysate
+ - + -
Lysosomes
- + - +
Cell lysate
+ - + -
Lysosomes
- + - +
LAMP1
100KDa
GAPDH
ATP6VoD2
50KDa
37KDa

Figure S1 Ai)

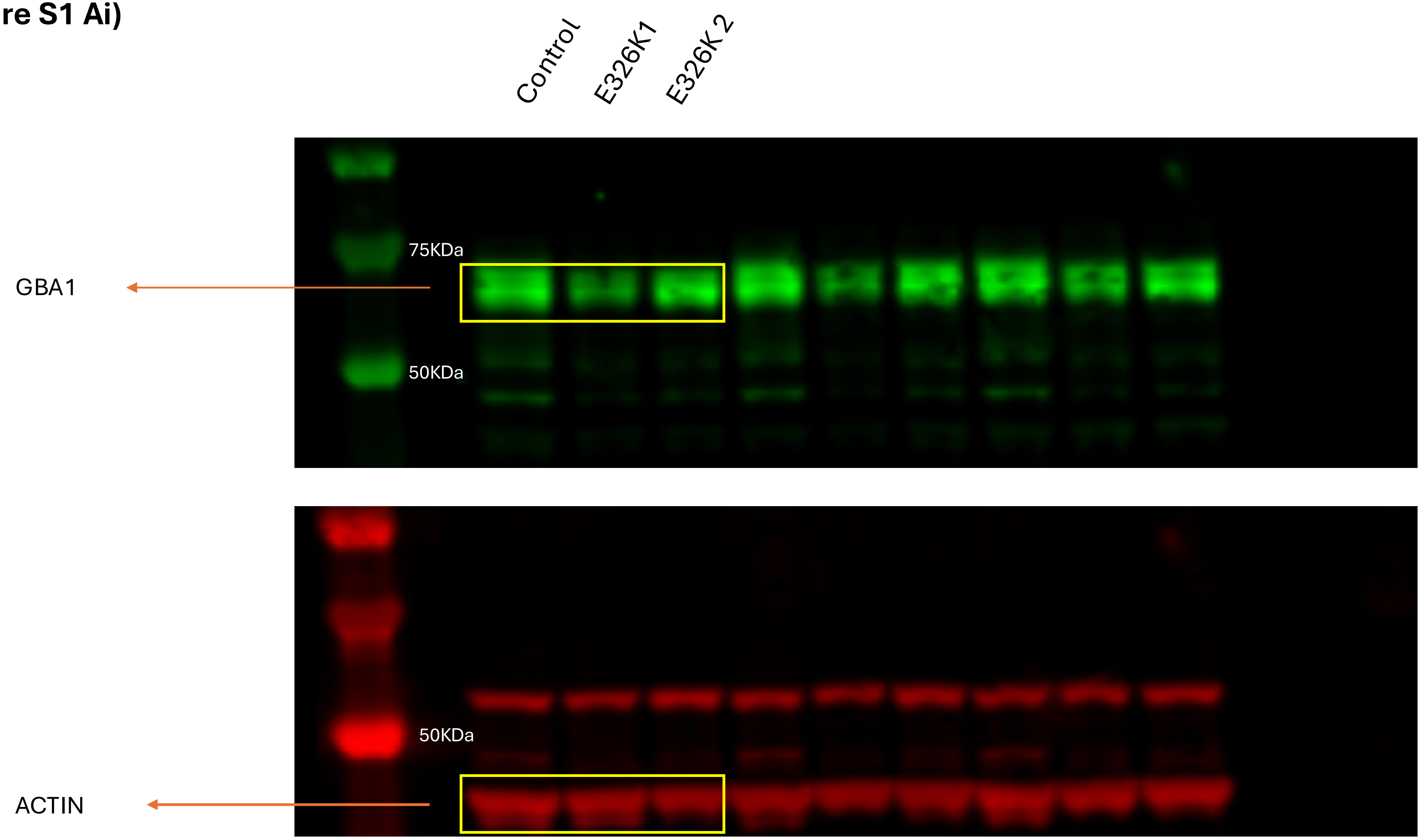

Figure S1 Ai)

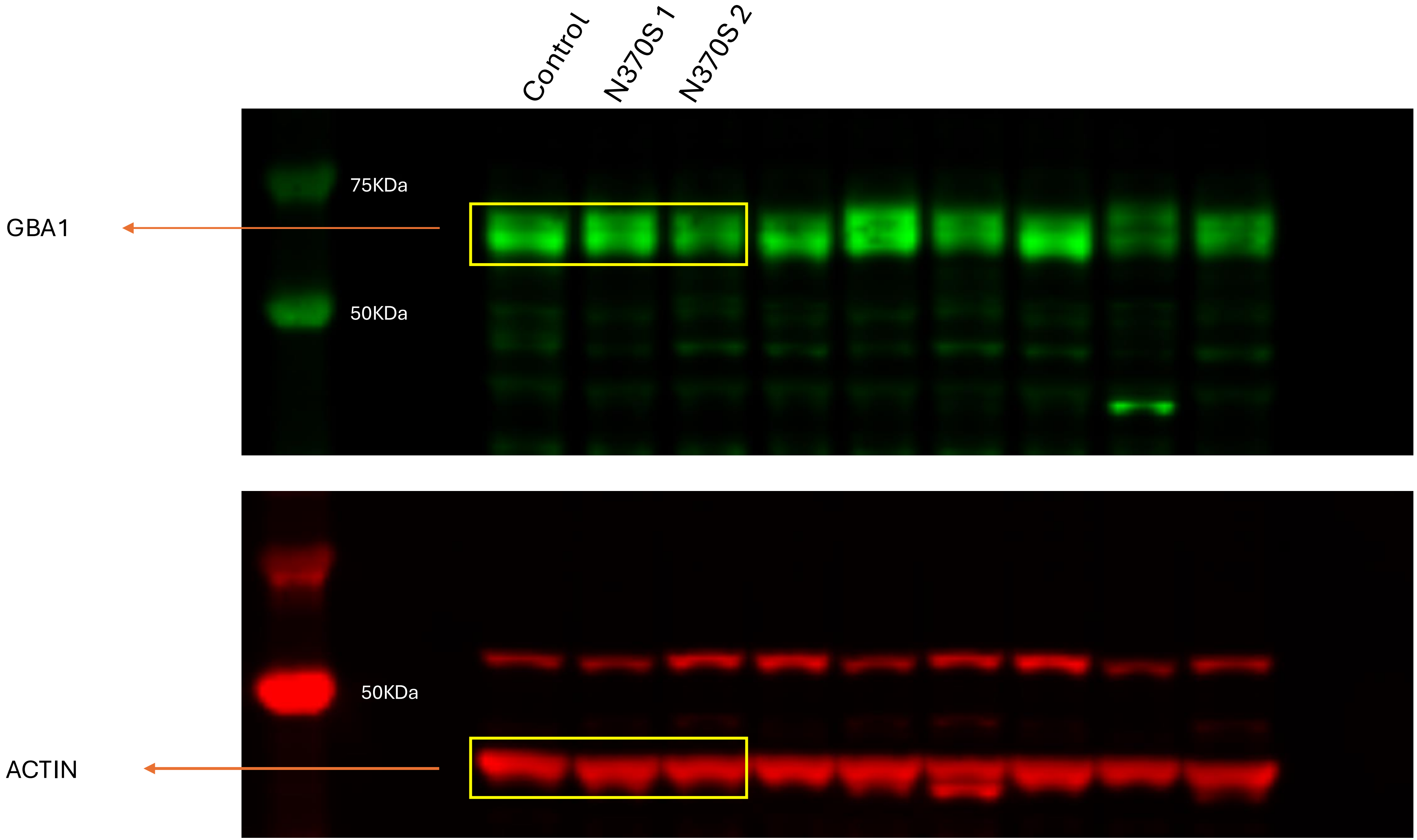

Figure S2 Di)

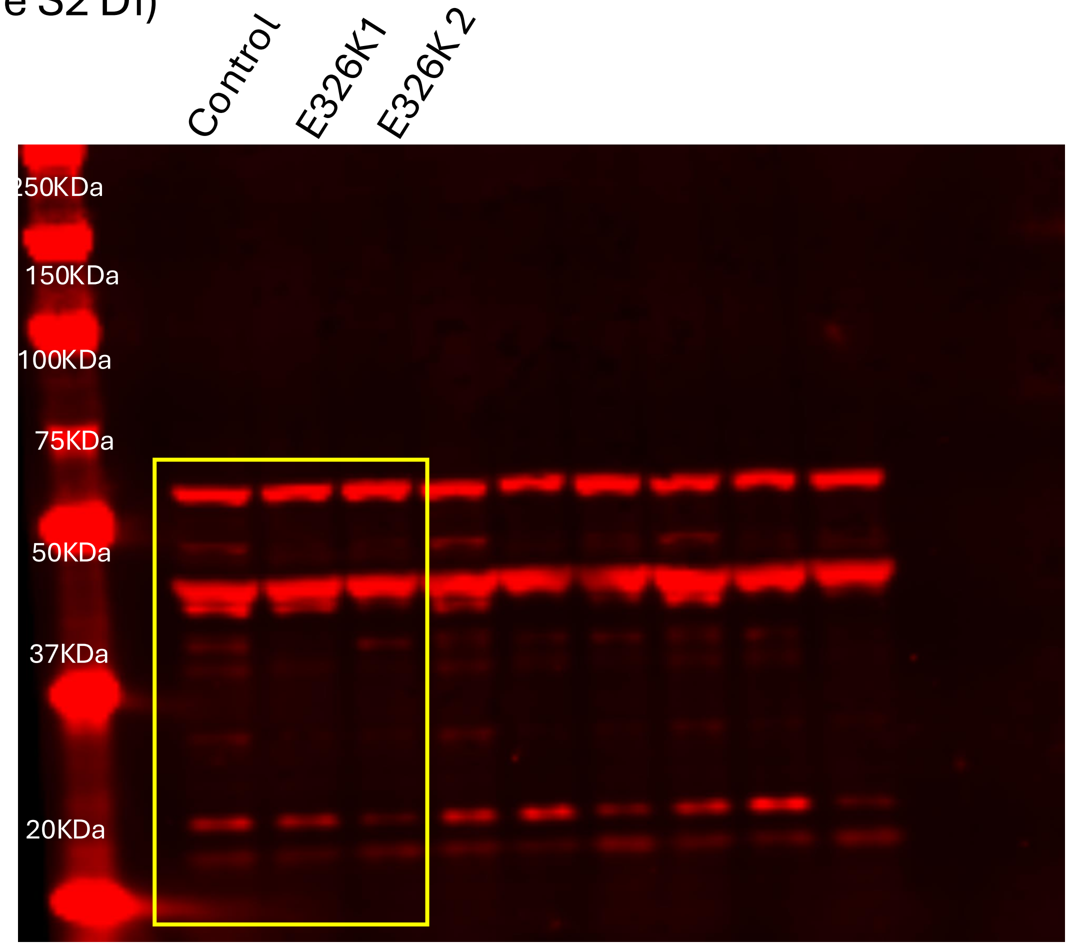

OXPHOS Complexes

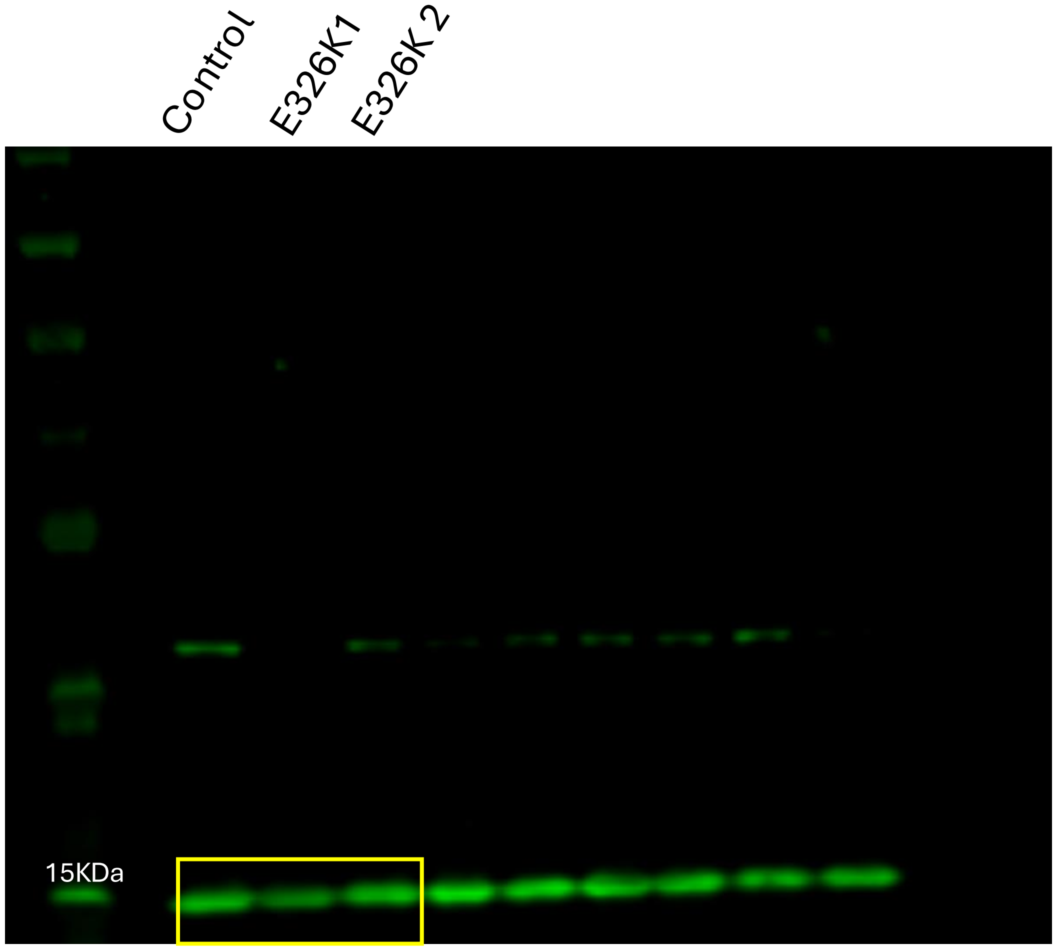

TOM20

Figure S2 Di)

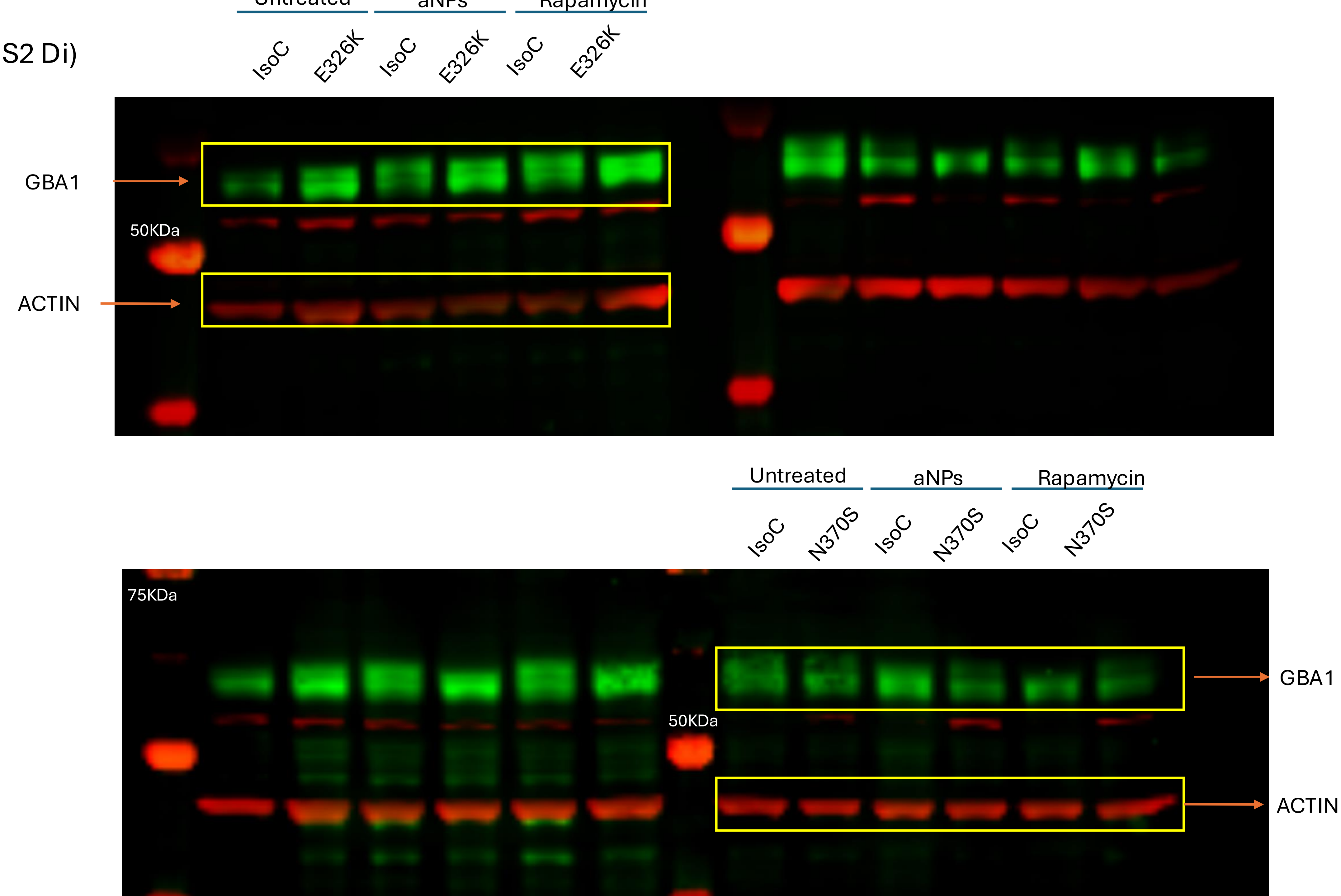
IsoC
E326K
IsoC
E326K
IsoC
E326K
GBA1
50KDa
ACTIN
Untreated
aNPs
Rapamycin
IsoC
N370S
IsoC
N370S
IsoC
N370S
75KDa
50KDa
GBA1
ACTIN

Figure S2 Ii)

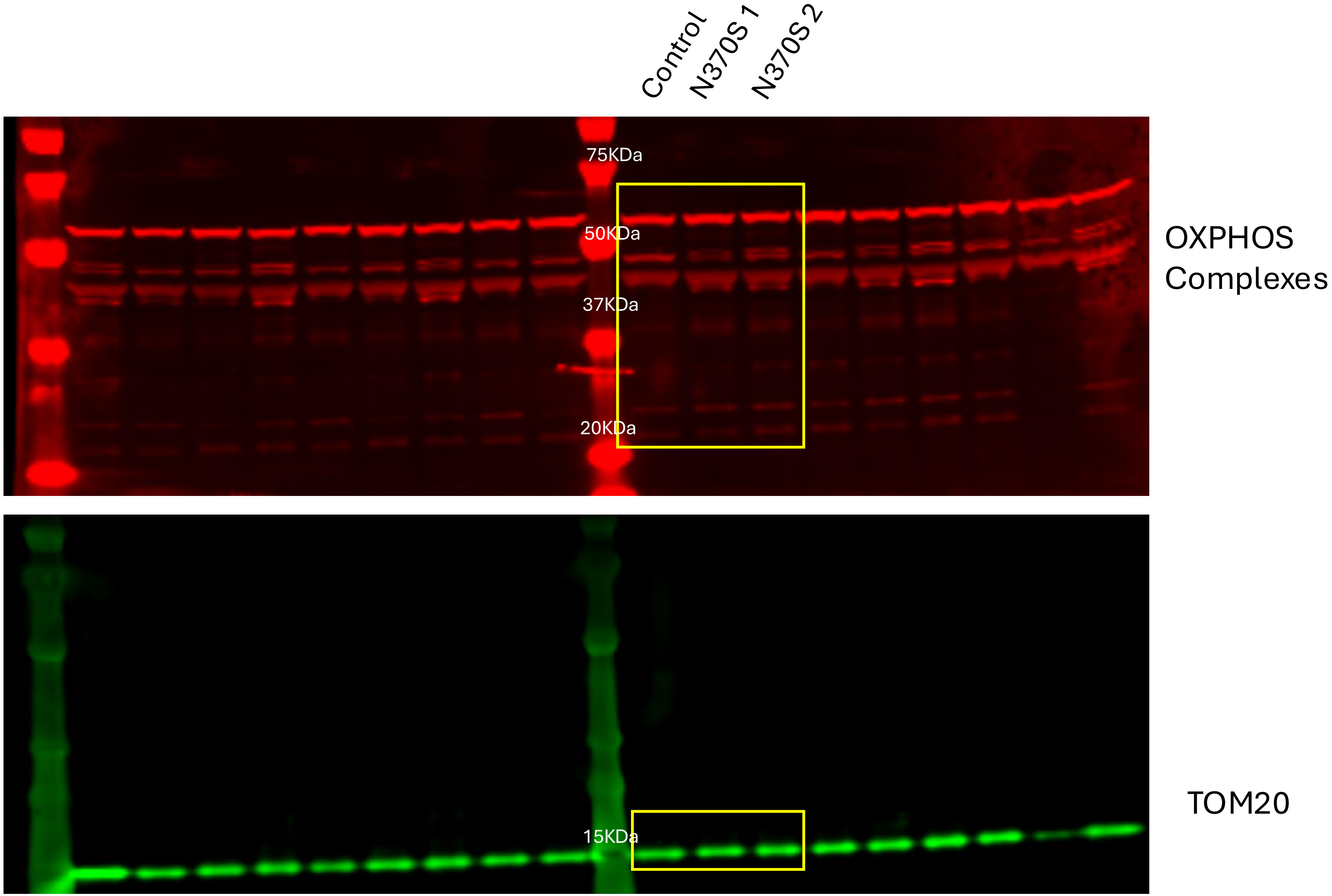

Control
N370S 1
N370S 2
75KDa
50KDa
37KDa
20KDa
OXPHOS Complexes
15KDa
TOM20

Figure S4 Ii)

OXPHOS Complexes

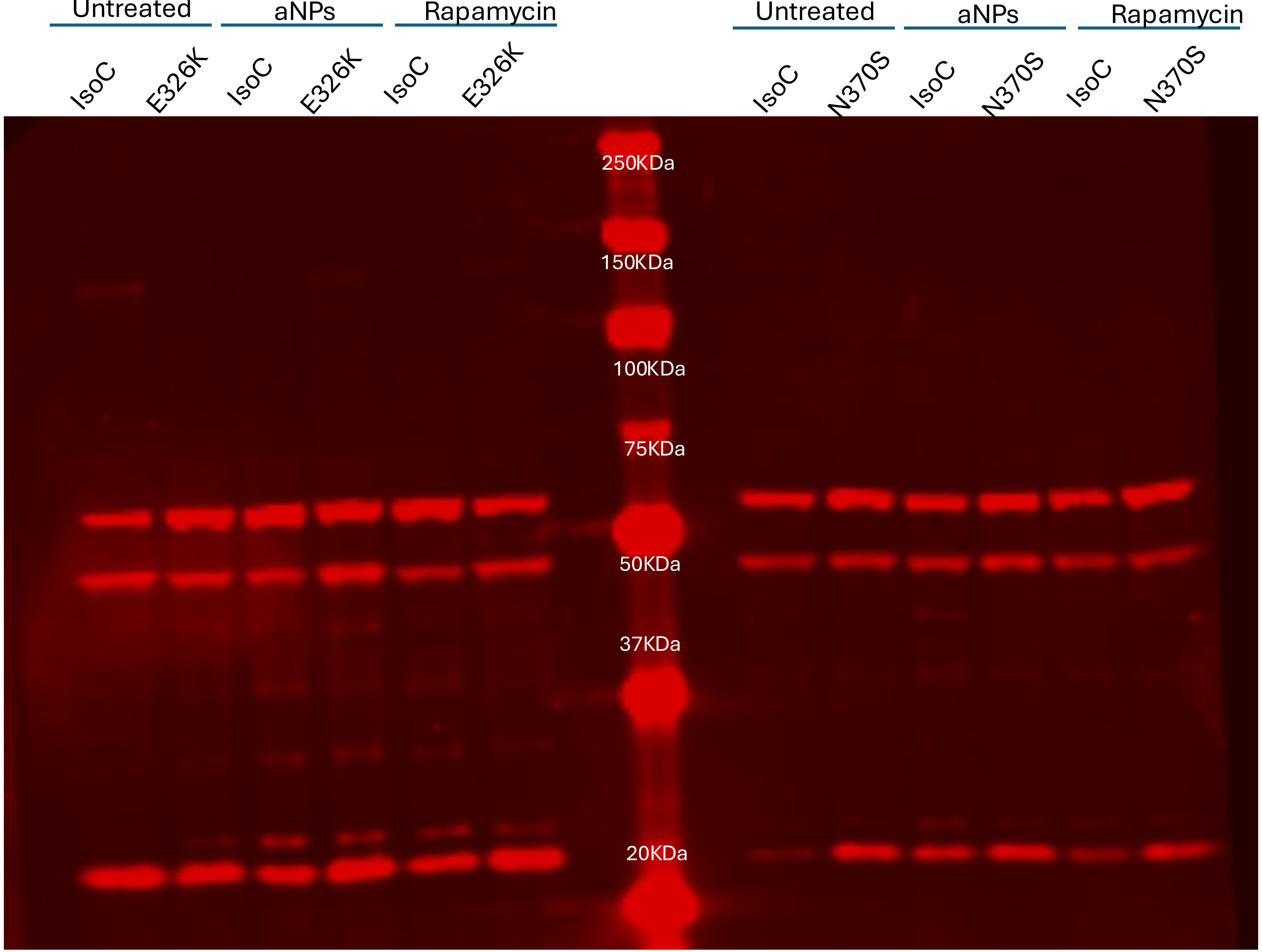

Figure S4 Ii)

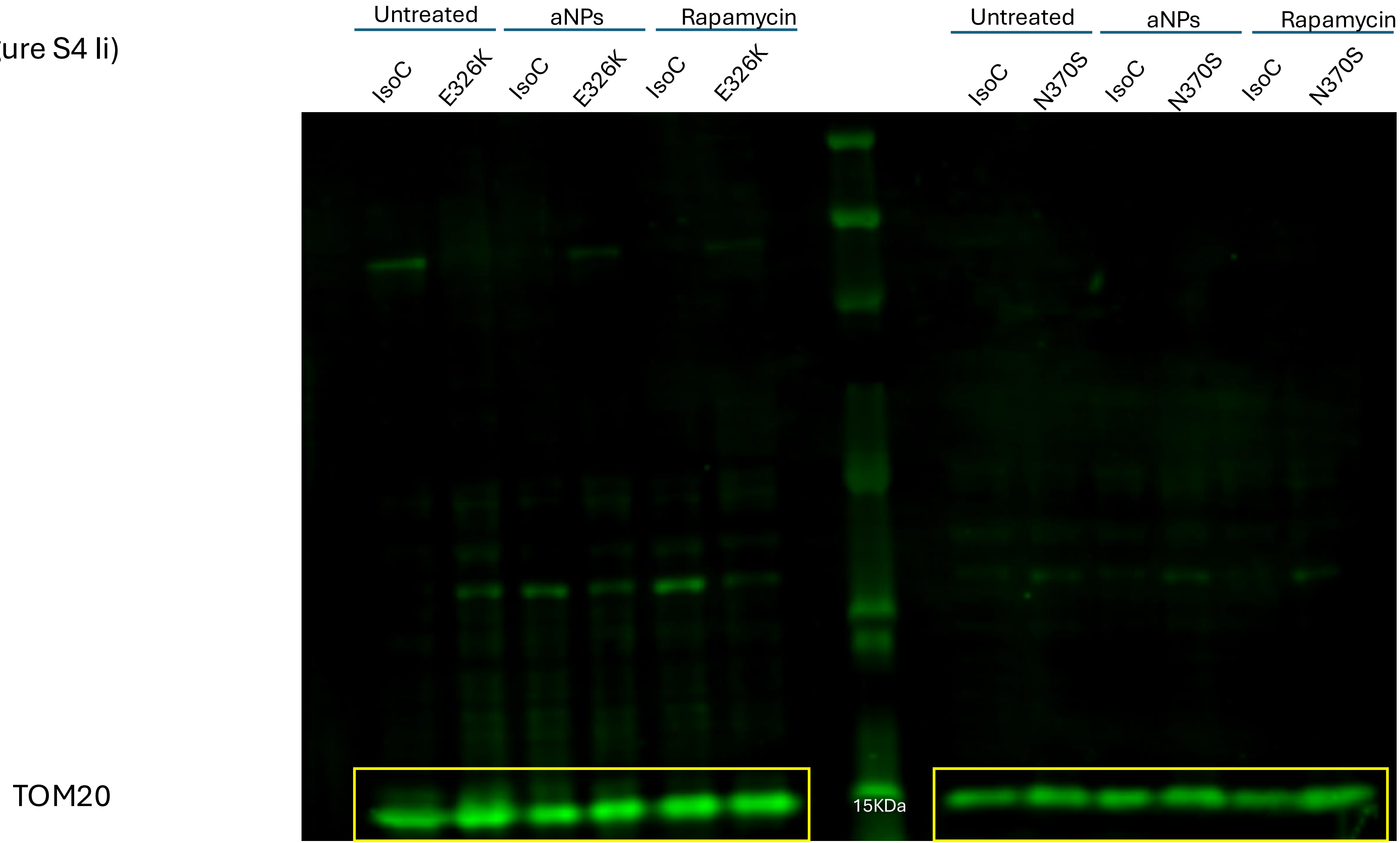
Untreated
aNPs
Rapamycin
Untreated
aNPs
Rapamycin
IsoC
E326K
IsoC
E326K
IsoC
E326K
IsoC
N370S
IsoC
N370S
IsoC
N370S
TOM20
15KDa

Figure 3Bi

n1 – Used in the manuscript

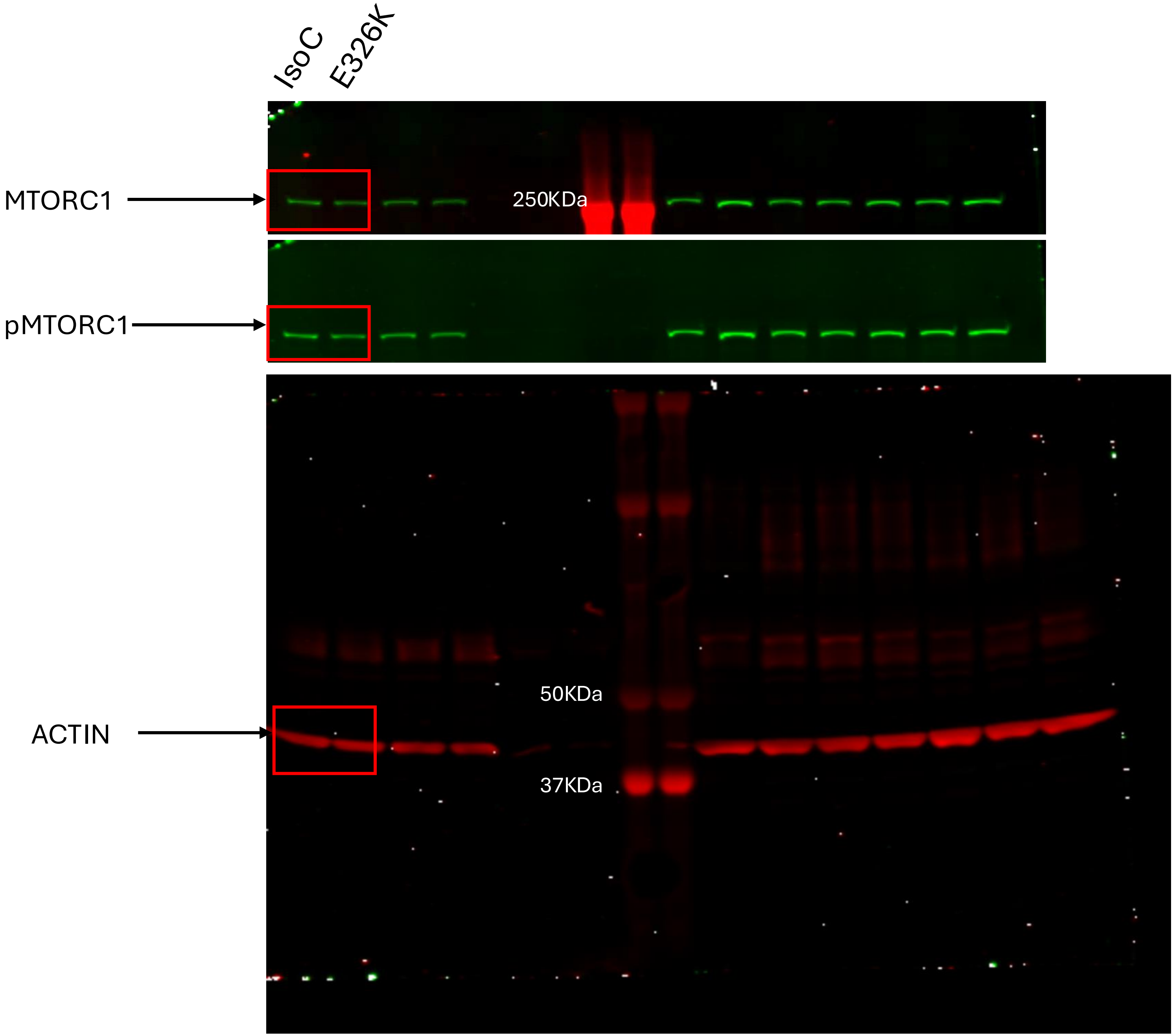

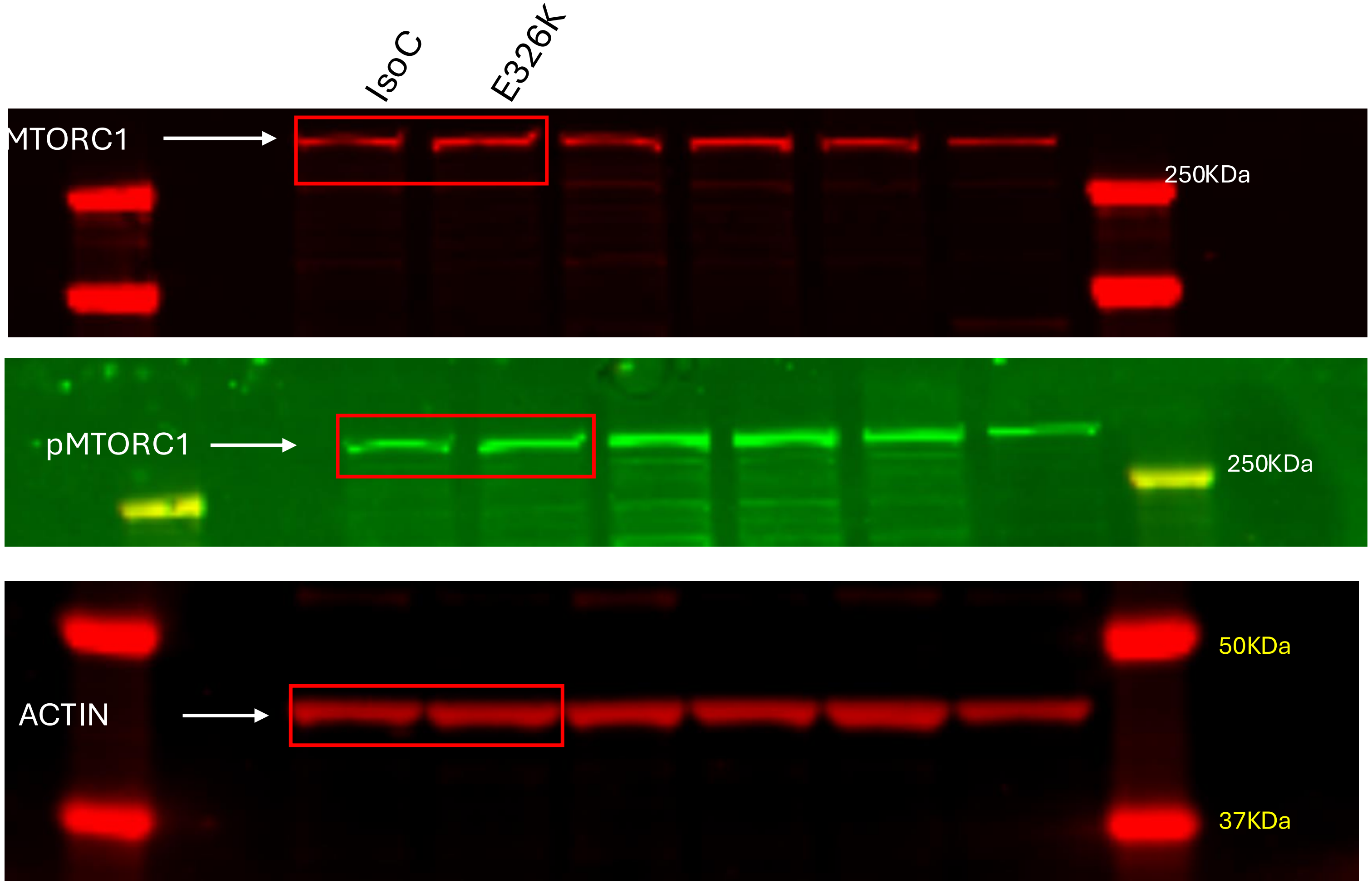

n2

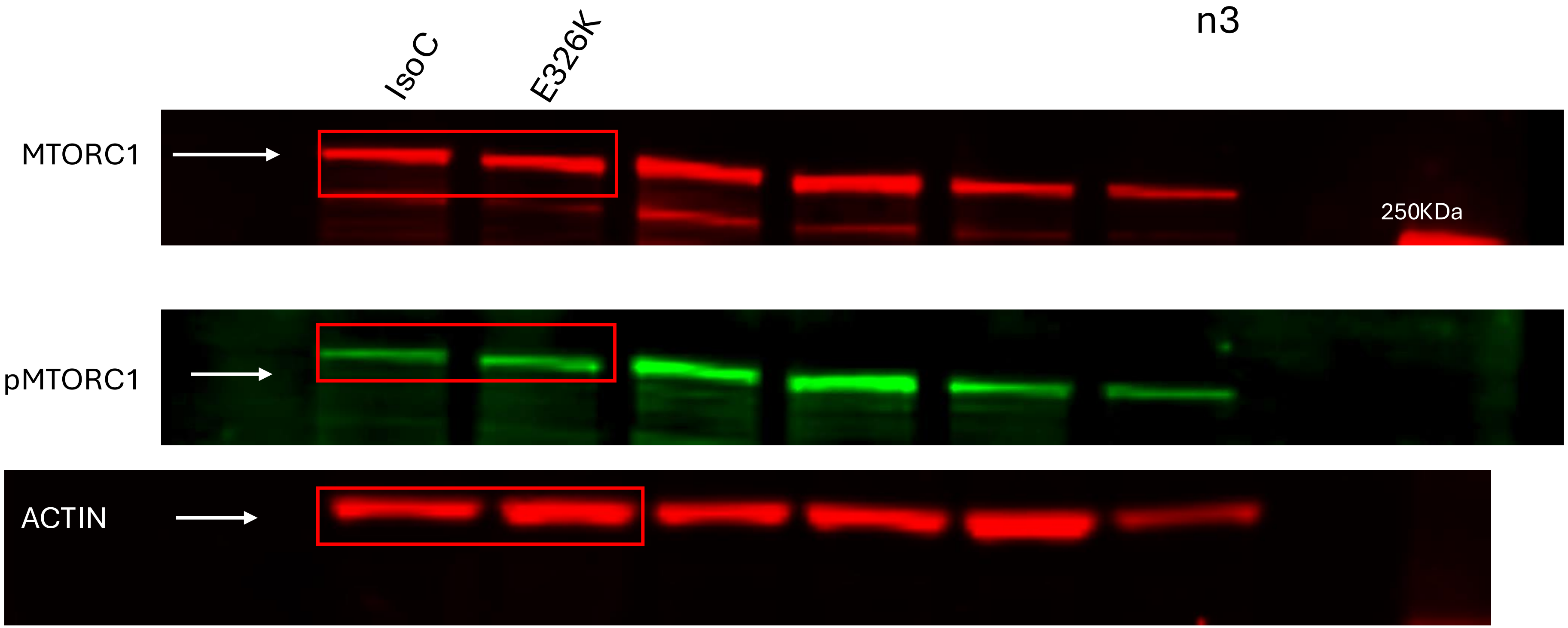

n3
IsoC
E326K
MTORC1
250KDa
pMTORC1
ACTIN

n4

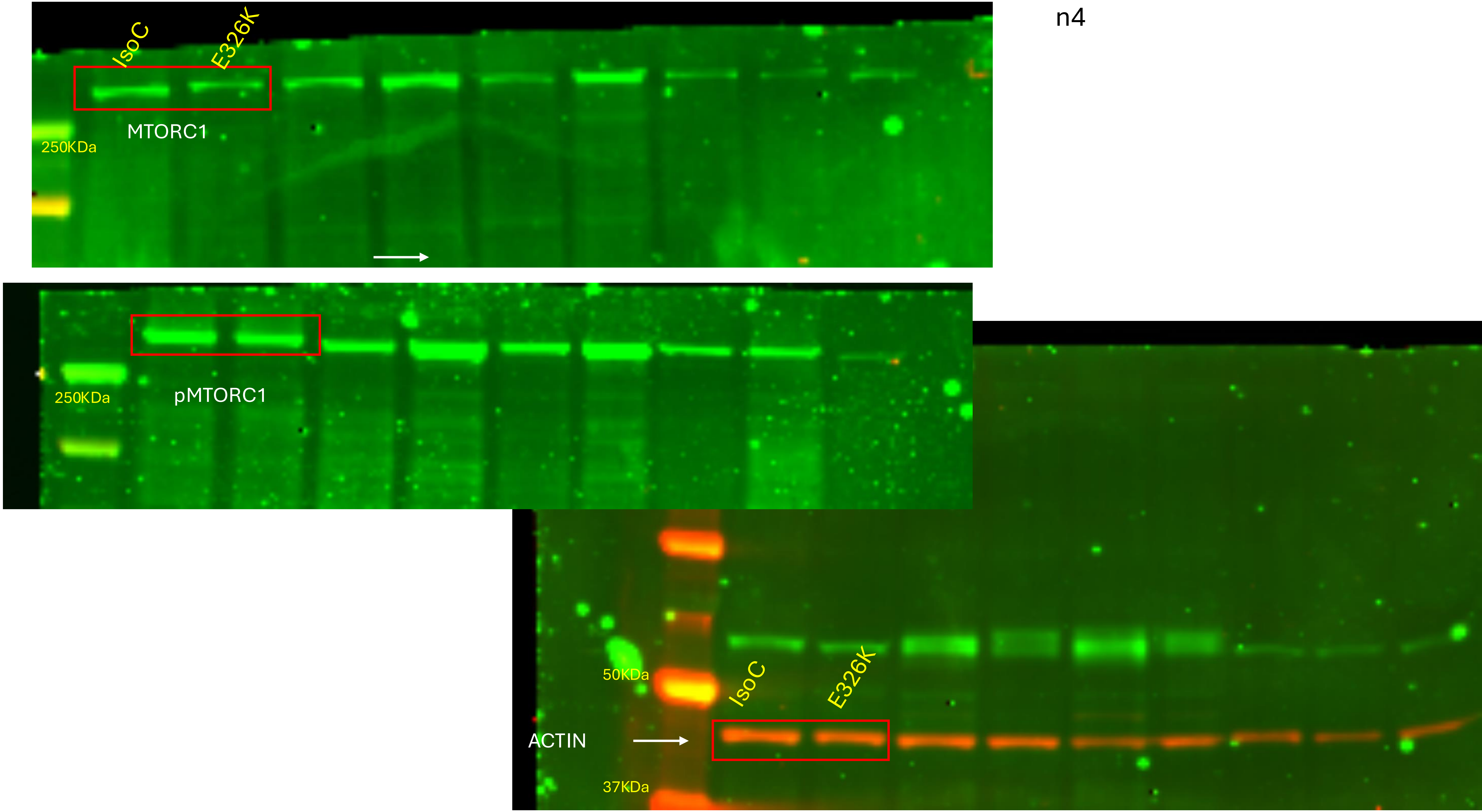
IsoC
E326K
MTORC1
250KDa
250KDa
pMTORC1
50KDa
IsoC
E326K
ACTIN
37KDa

Figure 3Bi

n1 – Used in the manuscript

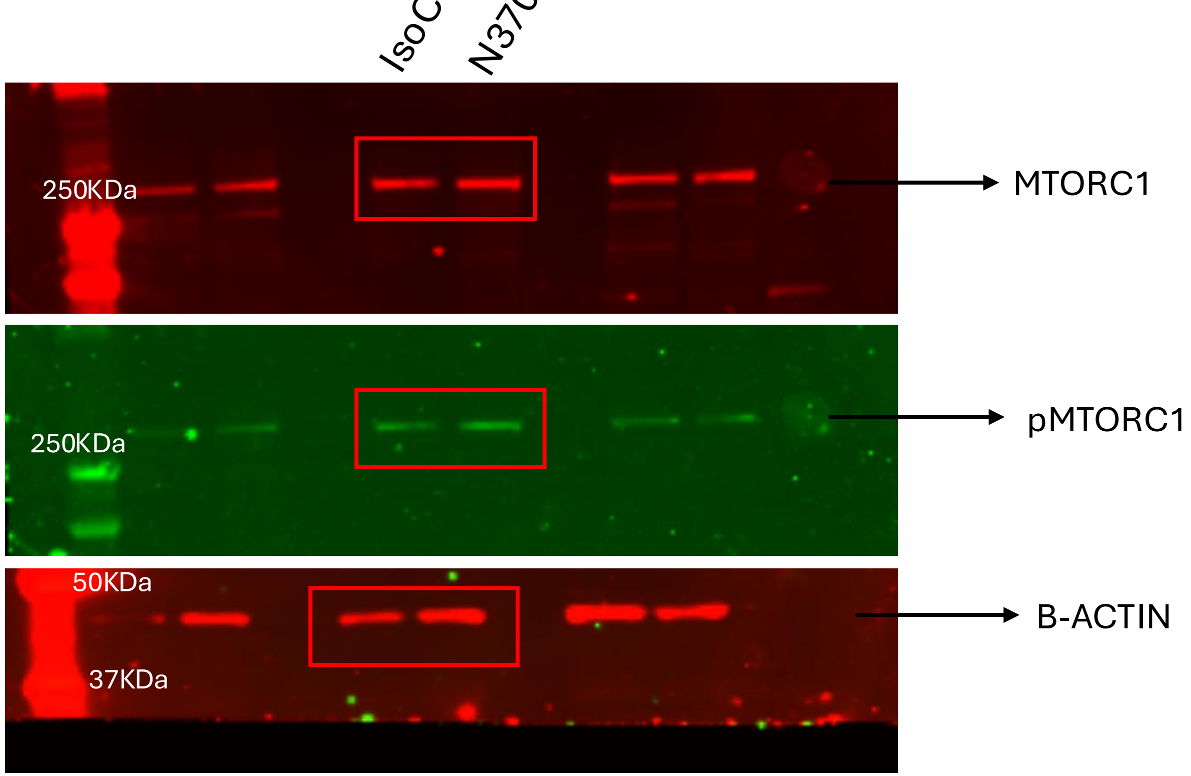

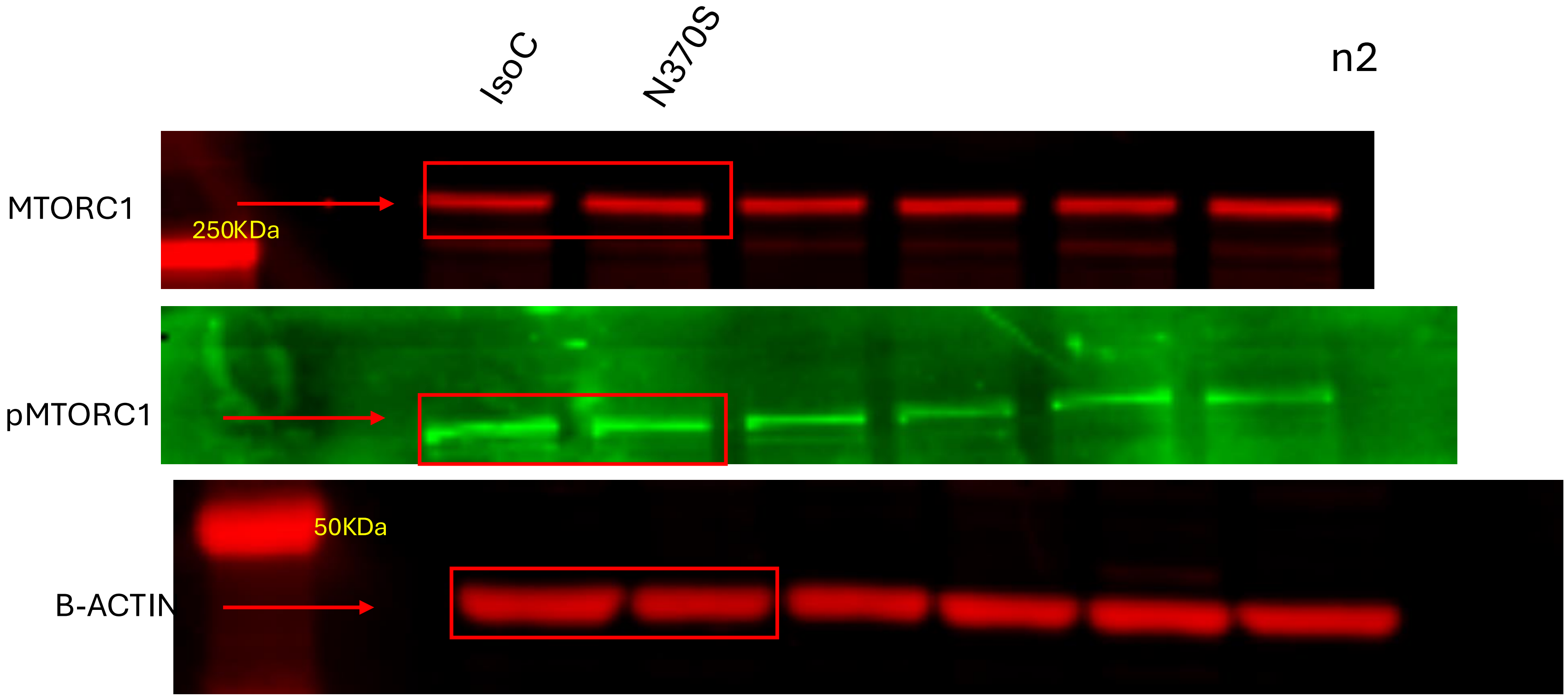
IsoC
N370S
n2
MTORC1
250KDa
pMTORC1
50KDa
B-ACTIN

n3

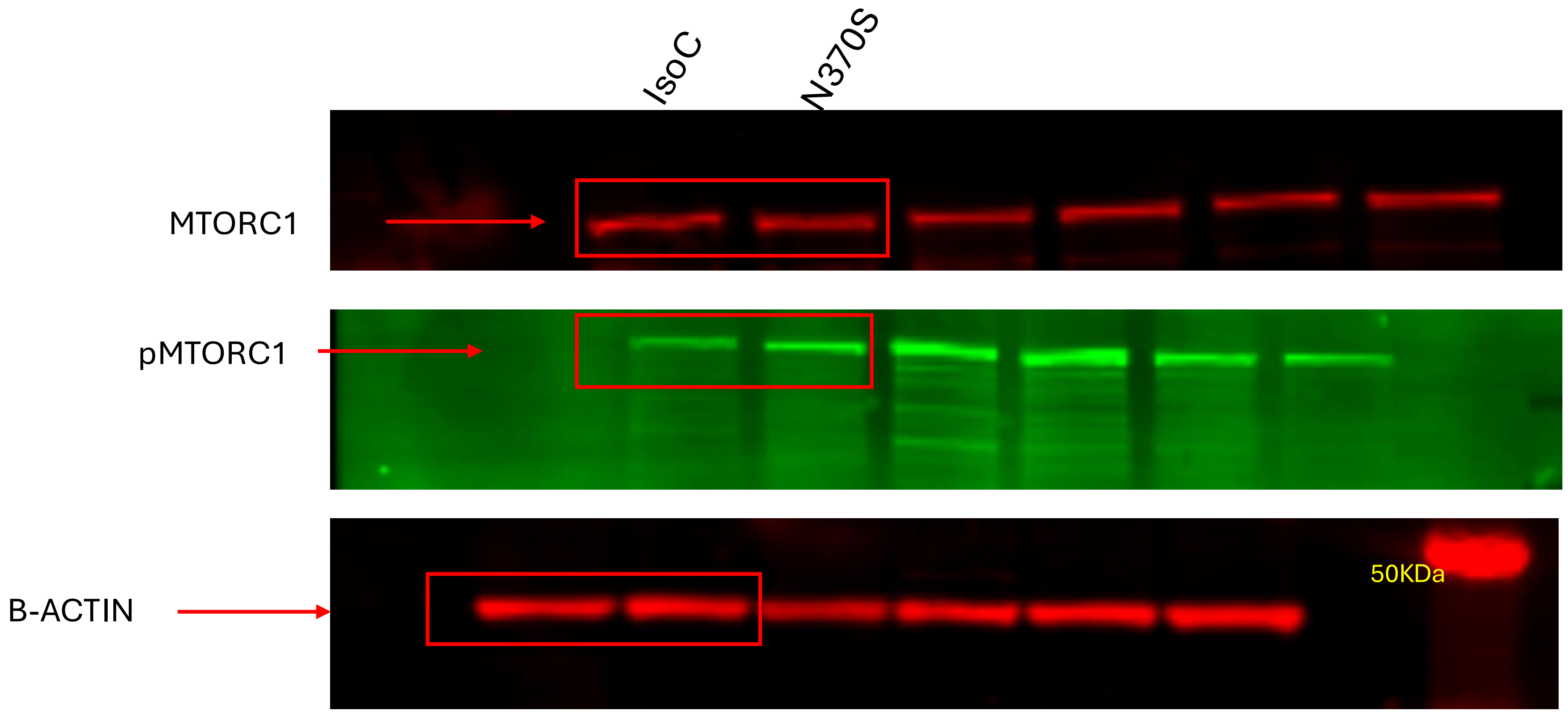

Supplement: Supplementary file 4 — Additional file 4. Original blots. [file 40035_2026_559_MOESM4_ESM.pdf]
